# Effects of Tobacco Smoking on the Degeneration of the Intervertebral Disc: A Finite Element Study

**DOI:** 10.1371/journal.pone.0136137

**Published:** 2015-08-24

**Authors:** Shady Elmasry, Shihab Asfour, Juan Pablo de Rivero Vaccari, Francesco Travascio

**Affiliations:** 1 Biomechanics Research Laboratory, Department of Industrial Engineering, University of Miami, Coral Gables, FL, United States of America; 2 Department of Neurological Surgery, The Miami Project to Cure Paralysis, University of Miami, Miami, FL, United States of America; Toronto Western Hospital, CANADA

## Abstract

Tobacco smoking is associated with numerous pathological conditions. Compelling experimental evidence associates smoking to the degeneration of the intervertebral disc (IVD). In particular, it has been shown that nicotine down-regulates both the proliferation rate and glycosaminoglycan (GAG) biosynthesis of disc cells. Moreover, tobacco smoking causes the constriction of the vascular network surrounding the IVD, thus reducing the exchange of nutrients and anabolic agents from the blood vessels to the disc. It has been hypothesized that both nicotine presence in the IVD and the reduced solute exchange are responsible for the degeneration of the disc due to tobacco smoking, but their effects on tissue homeostasis have never been quantified. In this study, a previously presented computational model describing the homeostasis of the IVD was deployed to investigate the effects of impaired solute supply and nicotine-mediated down-regulation of cell proliferation and biosynthetic activity on the health of the disc. We found that the nicotine-mediated down-regulation of cell anabolism mostly affected the GAG concentration at the cartilage endplate, reducing it up to 65% of the value attained in normal physiological conditions. In contrast, the reduction of solutes exchange between blood vessels and disc tissue mostly affected the nucleus pulposus, whose cell density and GAG levels were reduced up to 50% of their normal physiological levels. The effectiveness of quitting smoking on the regeneration of a degenerated IVD was also investigated, and showed to have limited benefit on the health of the disc. A cell-based therapy in conjunction with smoke cessation provided significant improvements in disc health, suggesting that, besides quitting smoking, additional treatments should be implemented in the attempt to recover the health of an IVD degenerated by tobacco smoking.

## Introduction

In the United States, approximately 20% of adults are cigarette smokers. Out of them, about 80% of individuals smokes every day [1]. The health consequences of tobacco consumption include multiple negative effects such as heart disease, multiple types of cancer, pulmonary disease, adverse reproductive effects, and the exacerbation of chronic health conditions [2]. In particular, epidemiological studies have demonstrated an association between smoking and low back pain [3–9].

Symptoms of low back pain have been strongly linked to the degeneration of the intervertebral disc (IVD) [10–13]. This degenerative process occurs upon disruption of the homeostatic balance between disc cell anabolism, responsible for the production of extracellular matrix (ECM), and cell catabolism, by which old ECM is removed from the IVD. *In vivo* experiments on animal models have shown how tobacco smoke exerts a detrimental effect on disc degeneration [14–19], and it has been hypothesized that this occurs via both direct and indirect pathways. For instance, *in vitro* tests showed that administration of nicotine, a major component of tobacco smoke, to IVD cell cultures reduces cell proliferation and biosynthesis of ECM components in a dose dependent fashion [20, 21]. Moreover, smoking causes: carboxy-hemoglobin production, which blocks oxygen transport in plasma [22]; vasoconstriction, which decreases the lumen of the vessels and reduces blood flow [22]; atheroma, which increases the thickness of arterial walls, thus decreasing blood flow [23]; impaired fibrinolytic activity, which reduces transvascular transport of nutrients to IVD [24]; and viscidation of the blood, which hinders oxygen transport [25]. Experimental studies on a porcine animal model showed that, upon acute smoking, a considerable constriction of the capillaries surrounding the IVD, and a marked reduction of oxygen and glucose levels in the nucleus pulposus (NP) occur [14]. Taken together, these results suggest that smoking may be responsible for IVD malnutrition, which is believed to be a leading cause of disc degeneration [26–28].

Investigating the implications of direct and indirect effects of smoking on IVD degeneration is challenging due to the complexity of the mechanisms regulating disc homeostasis. Moreover, through *in vivo* experiments, it may be difficult to differentiate the effects associated to direct exposure of disc tissue to nicotine from those due to impaired transport of nutrients because of smoking. Therefore, computational modeling is an alternative methodological approach for understanding and quantifying the effects of tobacco smoking on IVD homeostasis. Theoretical and numerical models have been increasingly used to investigate nutrition, cell viability, age-related disc degeneration, and transport of growth factors in the IVD [29–40]. The authors have developed a theoretical-computational framework describing IVD homeostasis which couples two factors regulating the levels of ECM components in the tissue: nutrients supply regulating cell viability and, indirectly, ECM production; and insulin-like growth factor-1 (IGF-1) availability, which mediates IVD cell proliferation, metabolism, and ECM biosynthesis [41]. In this study, this framework is deployed in an analysis aimed at understanding how and to which extent tobacco smoking can have detrimental effects on the homeostasis of the IVD. More specifically, the analysis hereby conducted examines: (1) to which extent nicotine can directly affect the biosynthesis of GAG and the proliferation of cells in IVD; (2) how determinant is malnutrition due to tobacco smoking in disrupting IVD homeostasis; and (3) if and to which extent the process of disc degeneration due to tobacco-smoking can be reverted by cessation of smoking alone or in conjunction with a cell-based therapeutic intervention.

## Methods

### Theoretical background

**Fig 1:**
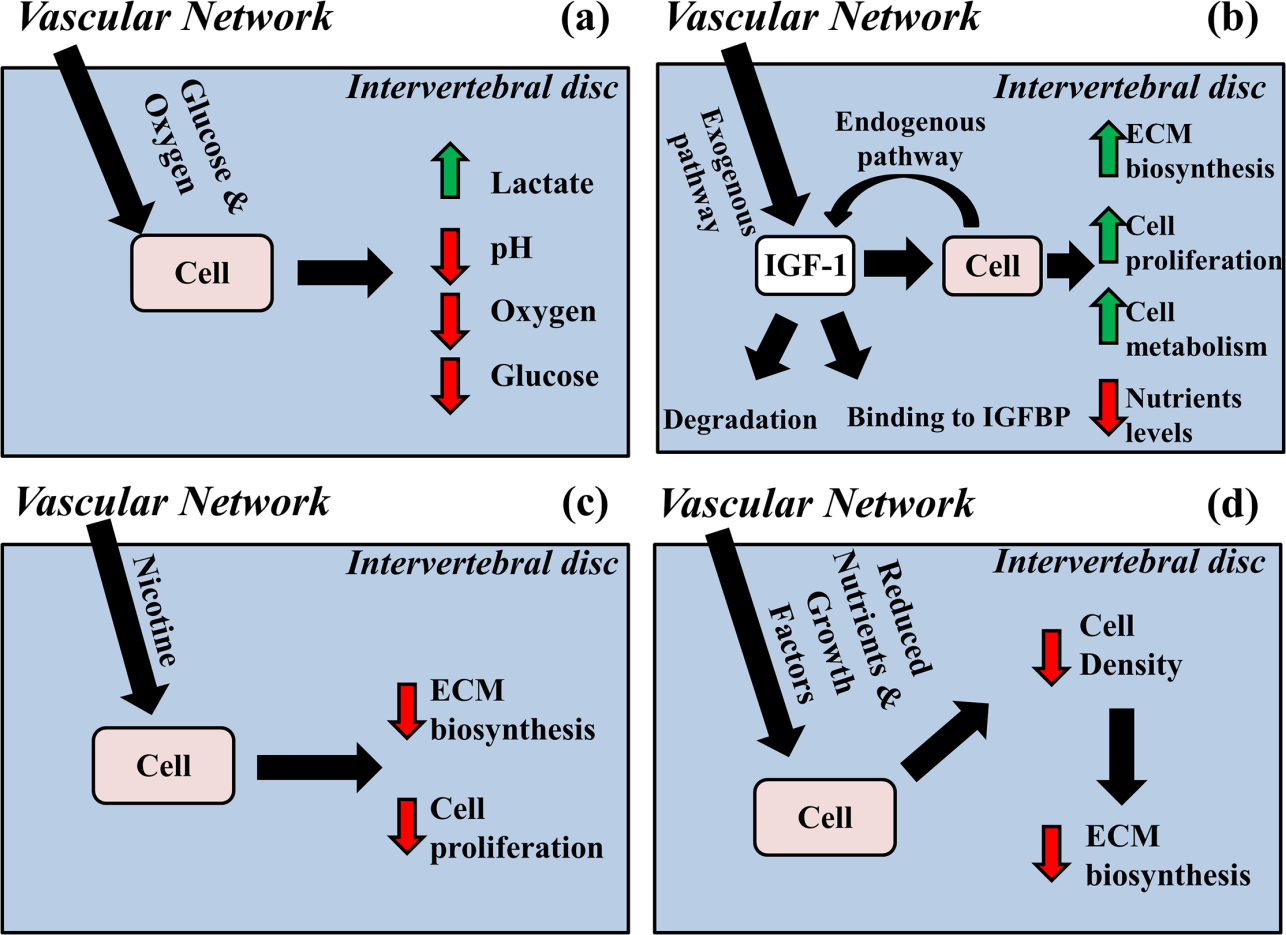
Schematic of the theoretical model of IVD homeostasis. (A) Metabolism of nutrients increases metabolites levels and reduces pH in the disc. (B) IGF-1 up-regulates cell proliferation and biosynthesis of ECM components. (C) Nicotine down-regulates cell proliferation and ECM biosynthesis (D) Tobacco smoking reduces solute supply to IVD causing reduction in cell density and ECM biosynthesis.

**Fig 2:**
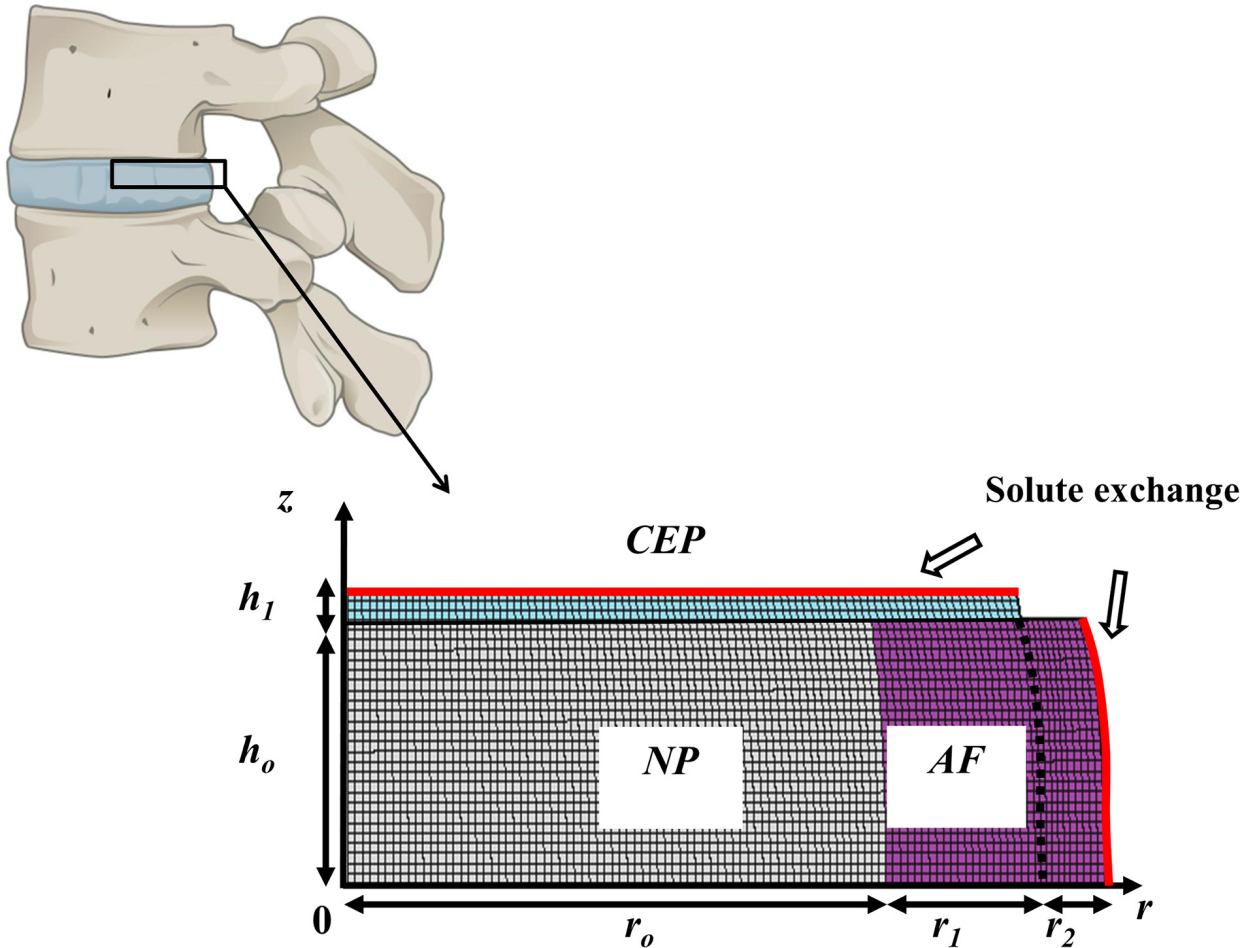
Computational domain. The IVD is confined between two vertebral bodies, and due to axial symmetry, only the right upper quadrant of IVD is modeled. The computational domain comprehends 3 regions: NP (gray), AF (purple), and CEP (light blue). Along the lateral surface of the AF and at the CEP, the disc is in contact with the vascular network providing solutes (i.e., nutrients, growth factor, binding proteins and nicotine). Sketch dimensions are *h*_*o*_ = 5.5 mm, *h*_*1*_ = 0.6 mm, *r*_*o*_ = 15.5 mm, *r*_*1*_ = 4.3 mm, and *r*_*2*_ = 2.0 mm.

A computational model was developed to investigate the effect of tobacco smoking on the homeostasis of the IVD. The model is based on a previously presented framework describing the homeostatic balance of the IVD through the diffusive-reactive transport of nutrients, metabolites and growth factors [41]. Nutrients (oxygen and glucose) duction of lactate. Adequate presence of nutrients in IVD is essential for cell viability since, when are transported into the IVD, and metabolized by disc cells with consequent pro glucose levels fall below critical values, cells die [26, 28, 42] (Fig 1A). The insulin-like growth factor-1 (IGF-1), upon binding to IGF-1 cell surface receptors (IGF-1R), increases in a dose dependent fashion the proliferation and the biosynthetic activity of IVD cells [43, 44]. IGF-1 is exogenously delivered from the vascular network surrounding the disc and endogenously produced by disc cells; its availability to IVD cells is regulated by a competitive reversible binding reaction of insulin-like binding proteins (IGFBP) (Fig 1B). The effect of tobacco-smoking on IVD homeostasis is accounted for in the model via two pathways: (1) nicotine diffusing in IVD from the surrounding vascular network reduces the rates of glycosaminoglycan (GAG) production and cell proliferation in a dose-dependent fashion [20, 21] (Fig 1C); (2) smoking causes an impaired transvascular transport of solutes, thus reducing the nutrient supply to IVD [14] indirectly affecting both cellularity and ECM biosynthesis (Fig 1D). A brief account of the model is reported in the ‘Appendix’. For a detailed description of the formulation of the model, the reader is referred to our previous works [29, 41].

### Finite element analysis

For computational purposes, the IVD was schematized as a two-dimensional (2D) domain with realistic anthropometric dimensions [36]. The NP and the inner annulus fibrosus (AF) were superiorly and inferiorly confined by cartilage endplates (CEP), while the outer AF was in contact with perfectly impermeable vertebral bodies. At the CEP and along the IVD lateral surface, the disc was in contact with the vascular network delivering solutes from plasma. Due to the axisymmetric property of the 2D computational domain, only the right upper quadrant of the IVD was modeled (Fig 2). Simulations were performed via commercially available finite element analysis software (COMSOL Multiphysics 4.4, COMSOL, Inc., Burlington, MA).

Model parameters regulating transport and chemical reactions of nutrients, metabolites, growth factor and binding proteins, together with rates of cell metabolism, IGF-mediated cell proliferation, and GAG production were the same as those utilized in our previous studies [29, 41]. The diffusivity of nicotine (*M*_*w*_^*N*^ = 162.23 g/mol) in the IVD is unknown, and its value was assumed to be proportional to that of glucose (*M*_*w*_^*Glucose*^ = 180.16 g/mol) according to the relation:

$$D^{N}=D^{Glu\cos e}\frac{M_{w}^{Glucose}}{M_{w}^{N}}.$$ (1)

**Fig 3:**
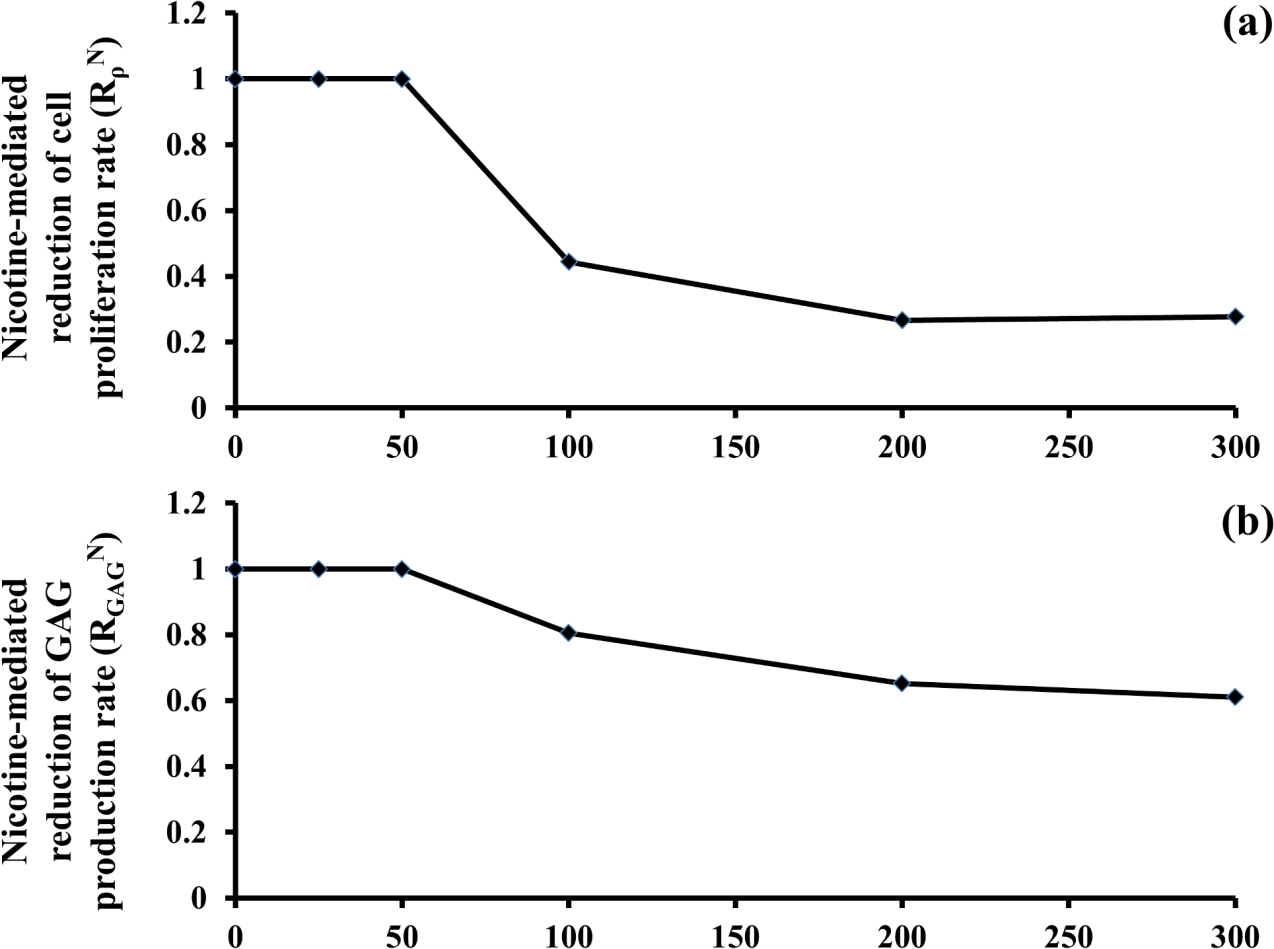
Nicotine-mediated down-regulation of cell anabolism and proliferation is dose-dependent (based on *in vitro* data [20]). (A) Nicotine-mediated reduction of cell proliferative rate. (B) Nicotine-mediated reduction of GAG production rate.

Moreover, the half-life of nicotine in IVD was 60 minutes [45]. Finally, the dose-dependent effects of nicotine on the reduction of cell proliferation ($R_{\rho }^{N}$) and GAG biosynthesis ($R_{GAG}^{N}$) were modeled as a piece-wise linear function of the nicotine concentration in the disc according to the experimental data provided in [20] (Fig 3).

Two pathways for disc degeneration due to tobacco smoking were simulated: (1) the nicotine-mediated down-regulation of GAG biosynthesis and cell proliferation; and (2) the reduction of solutes supply to IVD. The implications of both pathways on IVD homeostasis were investigated first separately (i.e., administrating nicotine and keeping normal levels of nutritional supply, or reducing nutritional supply with no nicotine), and then combined (i.e., administering nicotine while reducing the nutritional supply). Two scenarios were considered: ‘light smoking’ and ‘heavy smoking’. More specifically, when investigating the nicotine-mediate down-regulation of cell anabolism and proliferation, the concentration of nicotine at the boundaries of the disc was varied between 100nM (‘light smoking’ scenario) and 300nM (‘heavy smoking’ scenario) [46], while boundary concentrations of nutrients, growth factor, and binding proteins were set at the normal physiological values reported in the literature [30, 37, 38, 47]. The extent of reduction of solutes supply to IVD due to tobacco smoking is unknown. An *in vivo* study from Holm and Nachemson showed that prolonged tobacco smoking caused a reduction from 30% to 50% of oxygen and glucose concentrations in the NP of a porcine animal model [14]. Hence, when simulating the effect of a reduction of the solutes supplied to the IVD due to tobacco smoking, solutes boundary concentrations were deemed to attain oxygen and glucose concentrations in NP of about 70% (‘light smoking’ scenario) or 50% (‘heavy smoking’ scenario) of their normal physiological values. A summary of the boundary conditions used in this study is reported in Table 1. All the simulations were carried out over a time window sufficiently long to guarantee no significant changes over time in IVD for all the calculated quantities. Concentrations of glucose, oxygen, GAG, and cell density were averaged over disc regions (NP, AF, and CEP, respectively), and their values were normalized with respect to those attained at the ‘non-smoking’ scenario (i.e., normal physiological supply of nutrients and growth factor, and no nicotine).

**Table 1:** Boundary conditions of the IVD used in the finite element analyses.

Parameter	Value		Reference
	Periannular surface		
*Non-smoking scenario*			
*c* ^*Glucose*^		5 mM	[30]
*c* ^*O2*^		5.8 kPa	[37]
*c* ^*Lactate*^		0.9 mM	[37]
*c* ^*I*^		30.6 nM	[47]
*c* ^*BP*^		114.6 nM	[47]
*c* ^*BC*^		126.2 nM	[41]
*c* ^*N*^		0 nM	-
*Smoking scenario*	*Light*	*Heavy*	
*c* ^*Glucose*^	3.65 mM	2.15 mM	(*)
*c* ^*O2*^	4.23 kPa	2.5 kPa	(*)
*c* ^*Lactate*^	0.66 mM	0.39 mM	(*)
*c* ^*I*^	22.34 nM	13.16 nM	(*)
*c* ^*BP*^	83.66 nM	49.3 nM	(*)
*c* ^*BC*^	92.13 nM	54.3 nM	(*)
*c* ^*N*^	100 nM	300 nM	[46]
	Cartilage Endplate		
*Non-smoking scenario*			
*c* ^*Glucose*^		4 mM	[30]
*c* ^*O2*^		5.1 kPa	[37]
*c* ^*Lactate*^		0.8 mM	[37]
*c* ^*I*^		30.6 nM	[47]
*c* ^*BP*^		114.6 nM	[47]
*c* ^*BC*^		126.2 nM	[41]
*c* ^*N*^		0 nM	-
*Smoking scenario*	*Light*	*Heavy*	
*c* ^*Glucose*^	2.92 mM	1.72 mM	(*)
*c* ^*O2*^	3.72 kPa	2.2 kPa	(*)
*c* ^*Lactate*^	0.58 mM	0.34 mM	(*)
*c* ^*I*^	22.34 nM	13.16 nM	(*)
*c* ^*BP*^	83.66 nM	49.3 nM	(*)
*c* ^*BC*^	92.13 nM	54.3 nM	(*)
*c* ^*N*^	100 nM	300 nM	[46]

(*) Data obtained by deeming solutes’ boundary concentrations to attain oxygen and glucose concentrations in NP of about 70% (‘light smoking’ scenario) or 50% (‘heavy smoking’ scenario) of their normal physiological values [14].

## Results

### Effect of nicotine-mediated down-regulation of GAG biosynthesis and cell proliferation rate upon tobacco smoking on disc homeostasis

**Fig 4:**
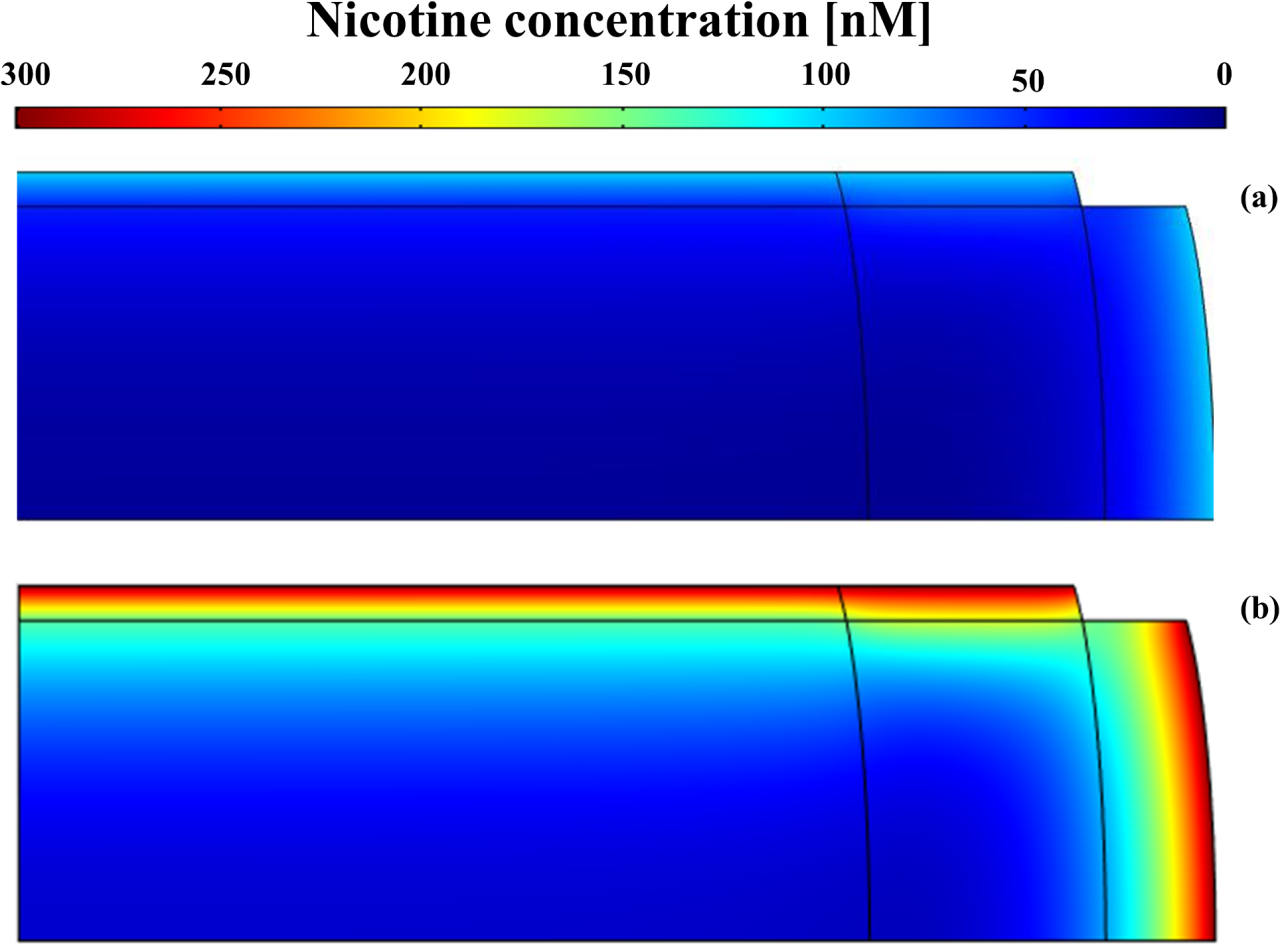
Nicotine distribution in the IVD. Data obtained assuming the boundary concentration of nicotine varying from 100nM to 300nM [46]: (A) nicotine boundary concentration of 100nM. (B) nicotine boundary concentration of 300nM.

Nicotine delivered from the vascular network surrounding the disc diffused throughout the entire tissue. The highest concentration levels were found at the periphery of the IVD (i.e., at the outer AF and at CEP) with values around 100 nM (‘light smoking’ scenario) and ranging from 200 to 300 nM (‘heavy smoking’ scenario) (Fig 4). In contrast, for both ‘smoking’ scenarios, the concentration in most of the inner IVD was less than 50 nM, a threshold level below which nicotine does not exert any down-regulating effect on disc cells [20] (Fig 3).

**Fig 5:**
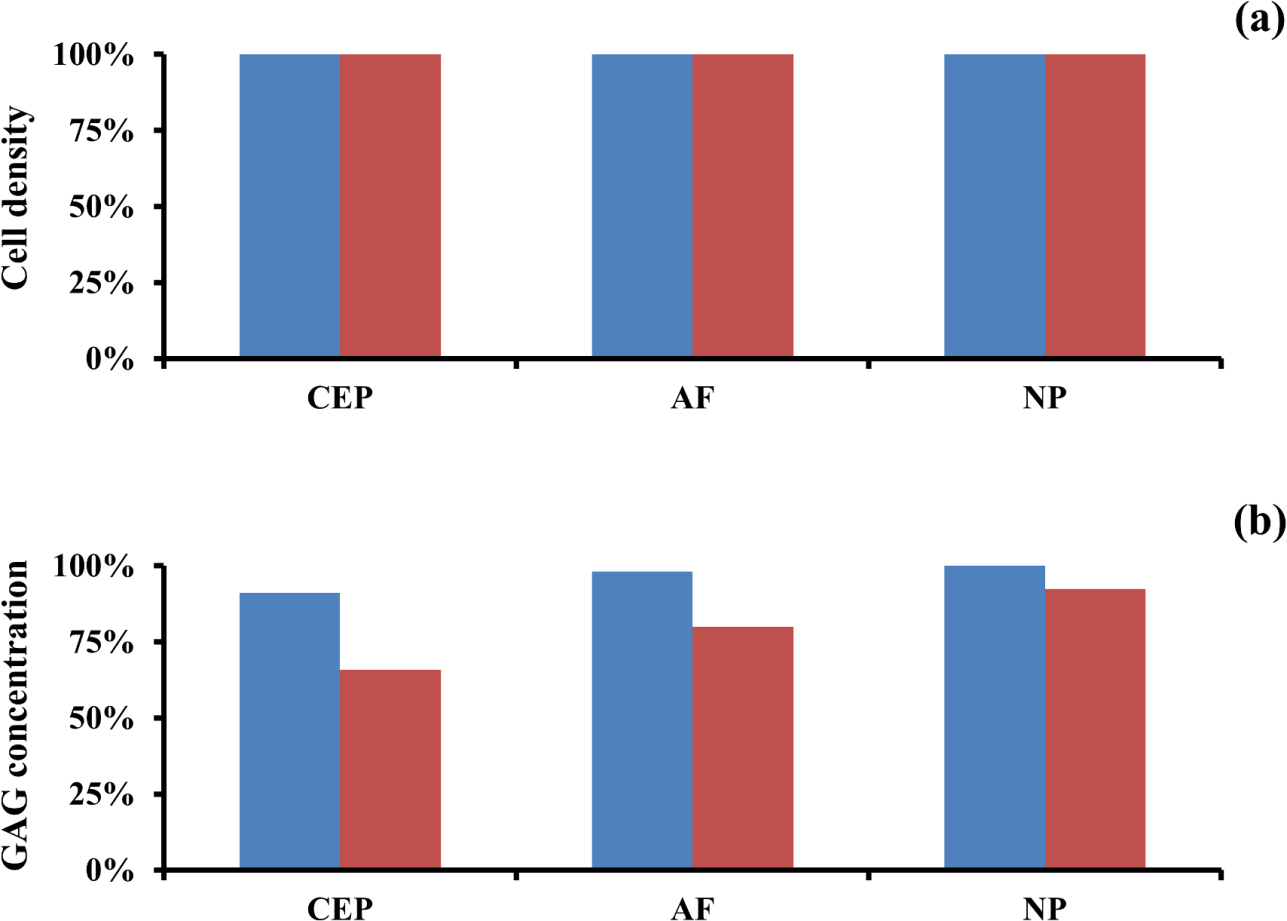
Effect of nicotine-mediated down-regulation of cell proliferation and GAG biosynthesis on IVD homeostasis. (A) Change in cell density in IVD regions. (B) Changes in GAG levels in IVD regions. Data are normalized with respect to the ‘non-smoking’ scenario. Data in blue are obtained assuming a nicotine boundary concentration of 100 nM, those in red assuming a nicotine boundary concentration of 300 nM.

The presence of nicotine did not affect the cellularity in the IVD, since nutrient levels in the tissue were sufficiently high (normal physiological levels) to support a normal physiological cell density (Fig 5A and S1 File). However, nicotine affected GAG levels in the IVD. When compared to a ‘non-smoking’ scenario, the external disc regions were mostly affected with reductions of GAGs. Specifically, GAG levels at CEP reduced to 91% (‘light smoking’ scenario) and 65% (‘heavy smoking’ scenario), and those at AF decreased to 98% (‘light smoking’ scenario) and 80% (‘heavy smoking’ scenario). In contrast, GAG content in the NP was unaffected in the case of ‘light smoking’, and was only reduced to 93% when simulating the ‘heavy smoking’ scenario (Fig 5B and S1 File).

### Effect of reduced solutes delivery to IVD upon tobacco smoking on disc homeostasis

**Fig 6:**
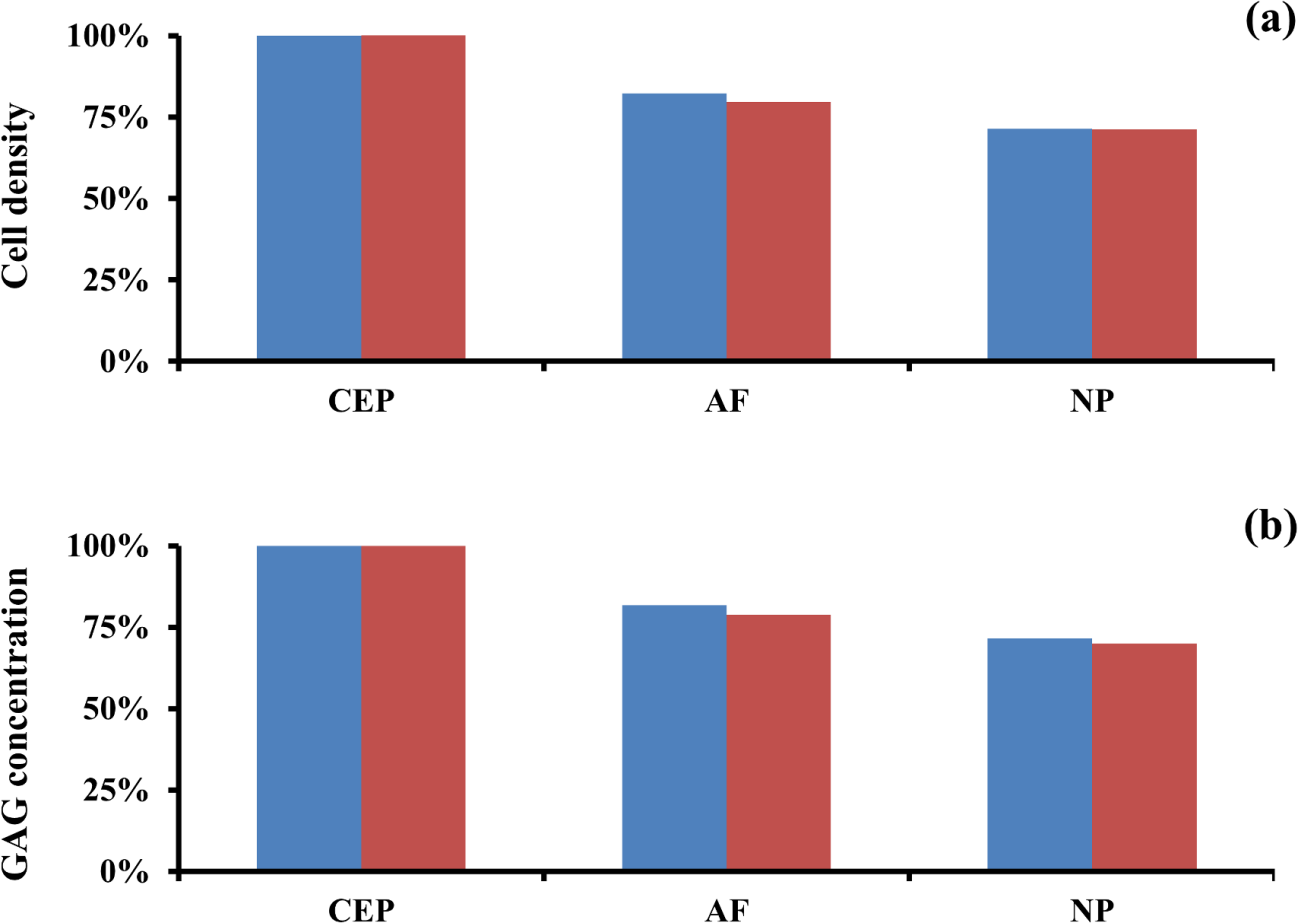
Effect of reduction of solute supply to IVD due to tobacco smoking on disc homeostasis. (A) Change in cell density in IVD regions. (B) Change in GAG levels in IVD regions. Data are normalized with respect to the ‘non-smoking’ scenario. Data in blue refer to ‘light smoking’ scenario, those in red to ‘heavy smoking’ scenario.

The reduction of nutrients and growth factor supply to disc tissue mostly affected the internal region of the IVD. For both smoking scenarios evaluated, cell density and GAG concentration in the NP were reduced to approximately 70% of those found in a ‘non-smoking’ scenario. The AF was affected in a similar fashion as the NP by the reduced supply of solutes. In contrast, at the CEP, cell density and GAG concentration remained at the normal physiological levels (Fig 6 and S2 File).

### Combined effects of nicotine and impaired solute delivery to the disc due to tobacco smoking on IVD homeostasis

**Fig 7:**
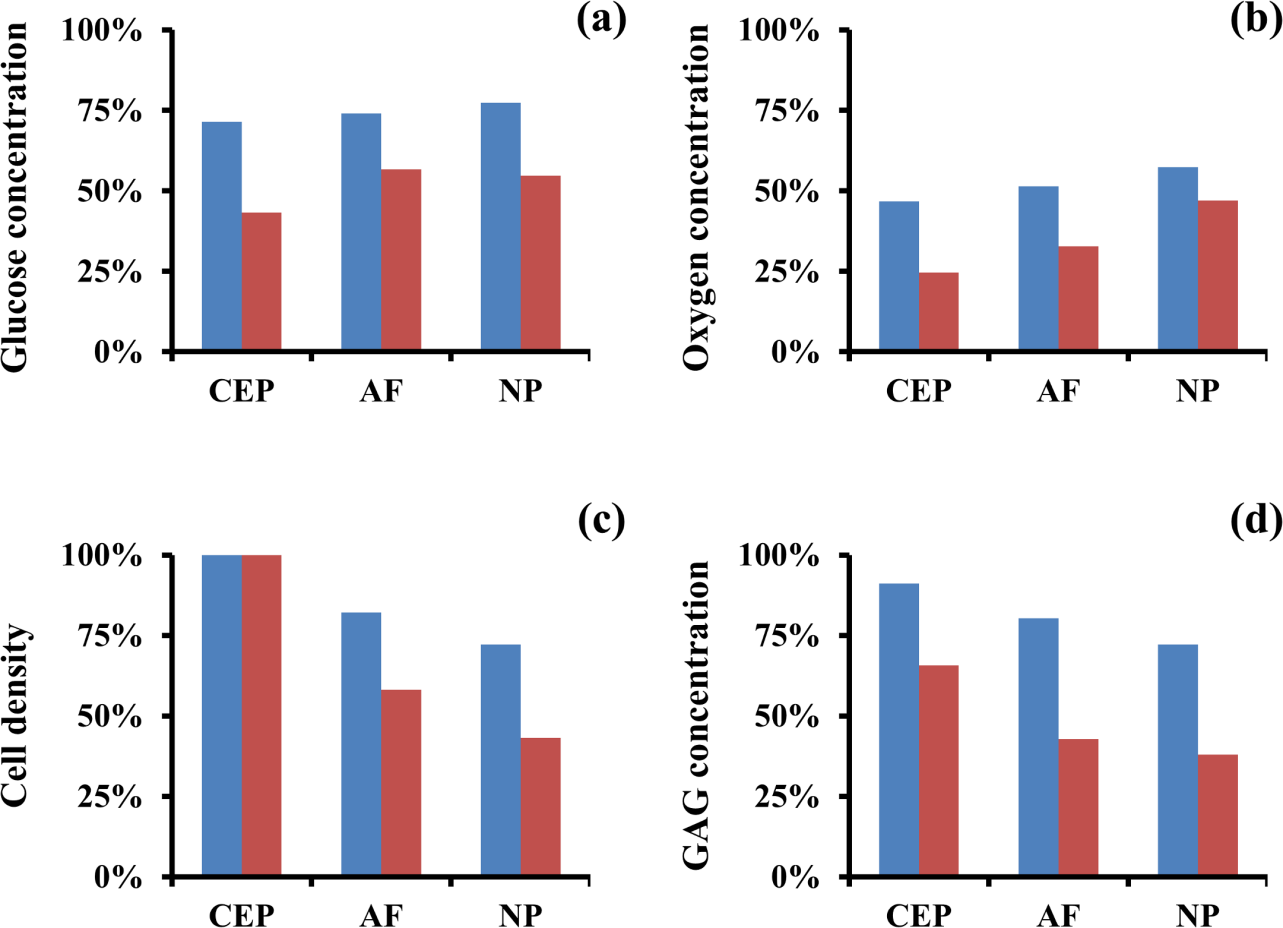
Effect of tobacco smoking on disc homeostasis. (A) Change in glucose concentration in IVD regions. (B) Change in oxygen concentration in IVD regions. (C) Change in cell density in IVD regions. (D) Change in GAG levels in IVD regions. Data are normalized with respect to the ‘non-smoking’ scenario. Data in blue refer to ‘light smoking’ scenario, those in red to ‘heavy smoking’ scenario.

The overall effect of smoking (nicotine presence and impaired solute supply) on the homeostasis of the disc was investigated. The reduction of nutrients supply to the IVD was reflected in the levels of glucose and oxygen observed in all disc regions. The largest reductions were found at the CEP, where glucose concentrations were 71% (‘light smoking’ scenario) and 45% (‘heavy smoking’ scenario) of those attained in a ‘non-smoking’ scenario, and those of oxygen were 47% (‘light smoking’ scenario) and 25% (‘heavy smoking’ scenario) (Fig 7A and 7B, and S3 File).

Despite the dramatic reduction in nutrient supply to the disc, the cell density at the CEP retained a normal physiological level for both smoking scenarios investigated. This occurs because, although reduced, glucose levels in CEP were still above those threshold values, causing cell death (0.2 mM [26, 28, 42]). In contrast, at the AF and the NP, the cell density decreased to 82% and 72% (‘light smoking’ scenario) and 58% and 43% (‘heavy smoking’ scenario) of normal levels, respectively (Fig 7C and S3 File). The content of GAGs decreased in all disc regions. The largest reductions were observed at the NP, approaching 72% (‘light smoking’ scenario) and 38% (‘heavy smoking’ scenario) of that attained in a ‘non-smoking’ scenario (Fig 7D and S3 File).

### Effect of quitting tobacco smoking and cell injection on the recovery of a degenerated IVD

**Fig 8:**
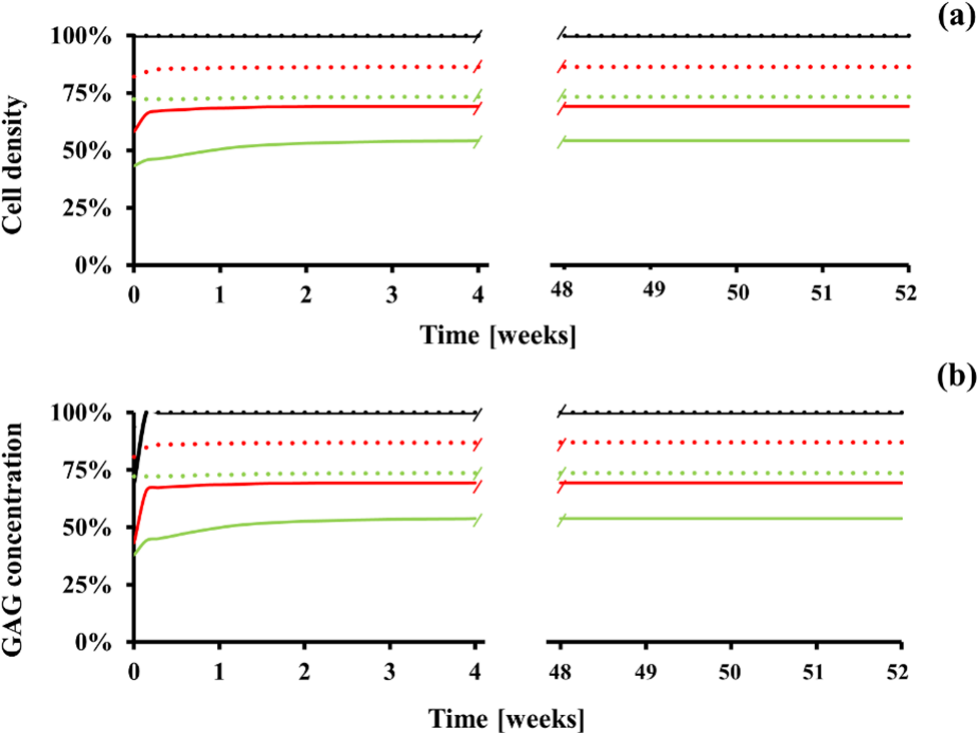
IVD homeostasis partially recovers at 1 year after quitting smoking. (A) Change in cell density. (B) Change in GAG concentration. Data are normalized with respect to the ‘non-smoking’ scenario and refer to the CEP (black line), the AF (red line), and the NP (green line). Dotted lines refer to ‘light smoking’ scenario, solid lines (red) to ‘heavy smoking’ scenario.

The effect of quitting tobacco smoking on the recovery of cell density and GAG levels in a degenerated IVD was also investigated. Quitting smoking was simulated by restoring the normal physiological supply of solutes and by eliminating nicotine delivery to a disc degenerated by tobacco smoking. Changes in cell density and GAG levels in the IVD were observed over the time frame of one year. For both smoking scenarios, cell density and GAG levels were significantly increased in all disc regions within few days after quitting smoking. Notably, the cell density and GAG concentration in the CEP fully recovered the normal physiological value within 1 day (Fig 8). In contrast, at the AF and the NP, cell density and GAG levels did not fully recover. At the end of the year, cell density in the AF and the NP were respectively 87% and 74% (light smoking), and 70% and 55% (heavy smoking) of those attained at normal physiological conditions (Fig 8A and S4 File); similar recovery patterns were observed for GAG levels (Fig 8B and S4 File).

**Fig 9:**
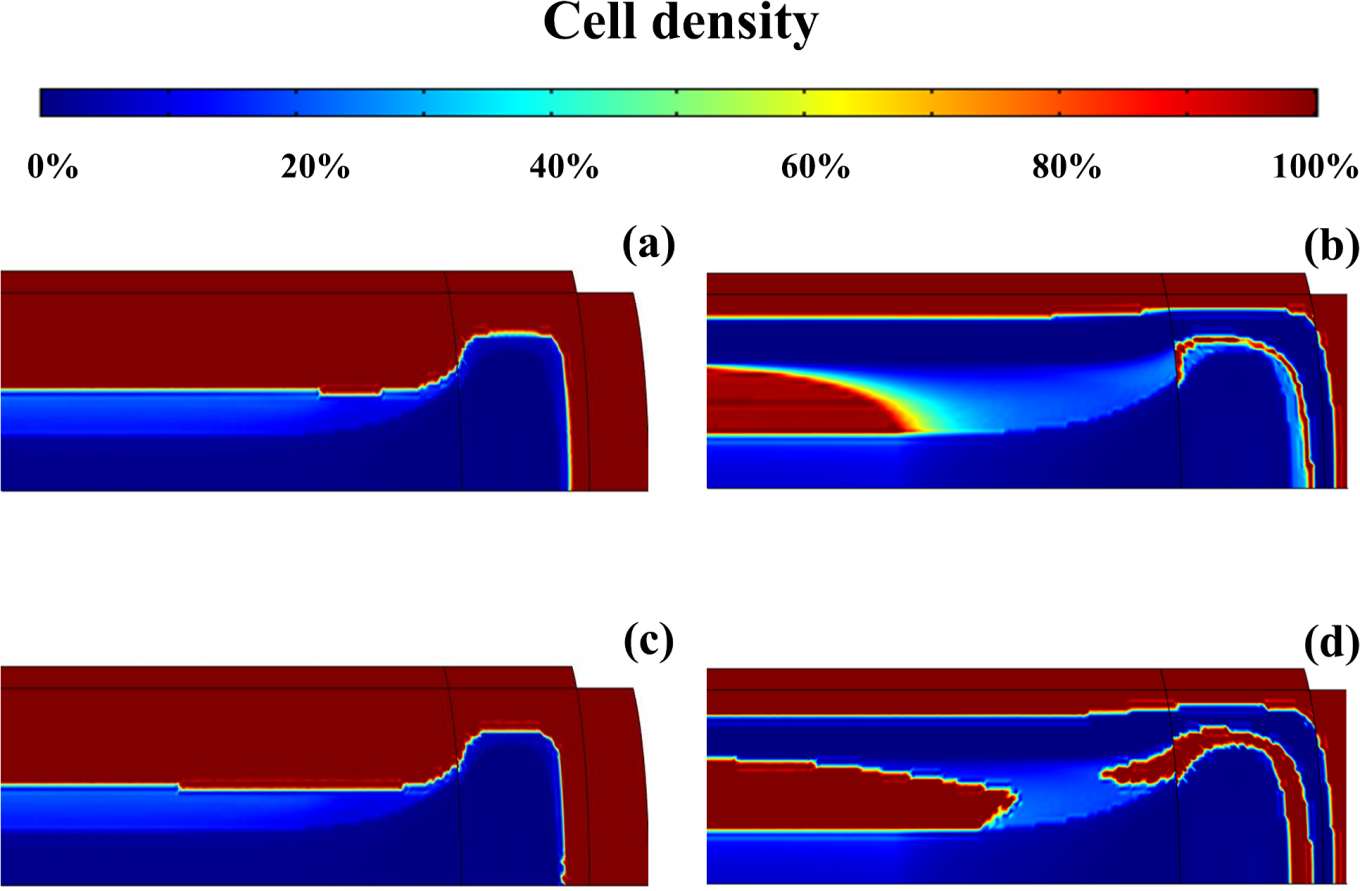
Cell density distribution in IVD changes after quitting smoking. (A) light smoker. (B) heavy smoker. (C) light smoker at 1 year after quitting smoking. (D) heavy smoker at 1 year after quitting smoking. Data are normalized with respect to the ‘non-smoking’ scenario.

The cell density distributions in both light and heavy tobacco smoking-degenerated IVD are compared to those attained after one year of quitting smoking (Fig 9). For the ‘light smoking’ scenario, cells are mostly present in the CEP, and in the outer AF and NP (Fig 9A). In contrast, for the case of ‘heavy smoking’, a region crossing the outmost NP and the middle AF presents poor cell density (Fig 9B). After quitting smoking, cellularity partially recovers in the inner NP (‘light smoking’ scenario), and along the middle of the NP and AF (heavy smoking) (Fig 9C and 9D). Minimal recovery was observed in those regions of the IVD which, due to tobacco smoking, were characterized by a cell density close to zero, such as the innermost NP and AF, and the NP close to CEP and in the region adjacent to the outer AF (only for heavy smoking). Similar changes were observed in the distribution GAGs in the IVD upon cessation of smoking (data not shown).

**Fig 10:**
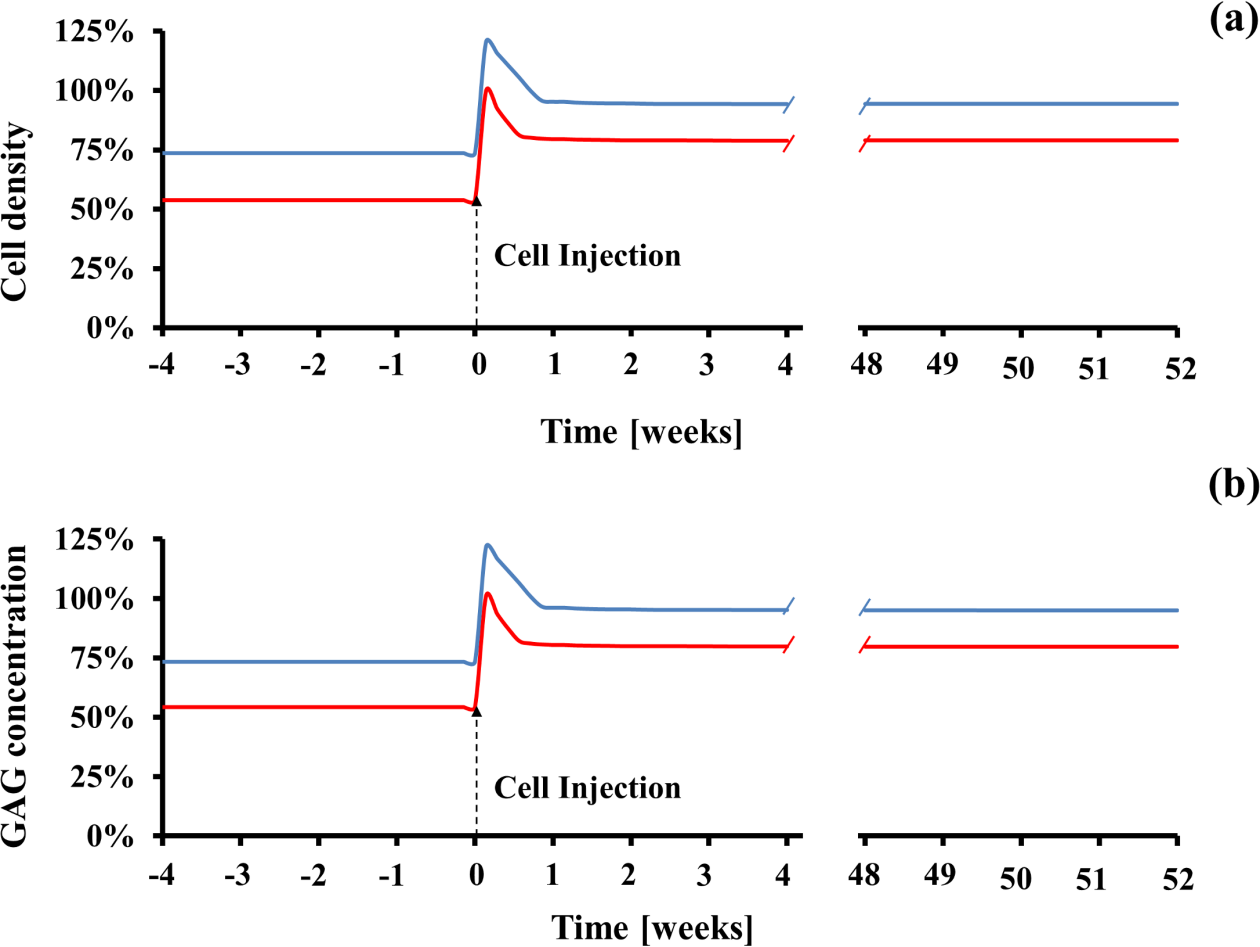
Injection of cells in the innermost NP improves disc homeostasis. (A) Change of cell density in NP. (B) Change of GAG concentration in NP. Data are normalized with respect to the ‘non-smoking’ scenario. Data in blue refer to ‘light smoking’ scenario, those in red to ‘heavy smoking’ scenario. For all the data reported, cell injection was performed one year after smoke cessation.

In order to improve cellularity and ECM composition in tobacco smoke-degenerated NPs, a cell-based therapy may be adopted. Accordingly, a stem cell injection in the NP after one year of smoke cessation was simulated. This was done by increasing the cell density up to 4200 cell/mm^3^ within a volume of 1350 mm^3^ in the innermost NP, under the assumption that injected stem cells would differentiate into NP cells. The effects of the cell injection on disc homeostasis were investigated over the time frame of one year (Fig 10 and S5 File). After a sudden post-injection increase, both cell density and GAG levels attained values of about 95% (light smoking) and 80% (heavy smoking) of those typical of normal physiological conditions. Such improvements in both cellularity and ECM composition of the NP were steadily kept during the one year period of observation.

## Discussion

The harmful effects of tobacco smoking have been investigated in every field of medicine. In particular, in the orthopaedic field, smoking has been associated with low back pain [6, 48], especially in the adolescent population [48–51]. Low back pain has been strongly linked to IVD degeneration [10–13]. Experimental studies on animal models have shown that nicotine, a major component of tobacco smoke, causes the degradation of the ECM of the disc [15–19]. However, the exact mechanisms by which tobacco smoke causes IVD degeneration are still unclear, and it has been hypothesized that: (1) nicotine, by exerting a down-regulation of disc cells proliferative rate and biosynthetic activity, destabilizes the homeostatic balance of the IVD, hence leading to disc degeneration [52]; (2) tobacco smoke exerts a persistent constriction of the vascular network around the disc, thus reducing transvascular transport of nutrients and causing malnutrition of the IVD, which is believed to be a leading cause of degeneration [26–28]. In this study, we adopted a computational model describing IVD homeostasis [41] to quantify the effects of nicotine-dependent down-regulation of cell anabolism and solute exchange impairment due to smoking on the health of the disc tissue.

The nicotine-mediated down-regulation of disc cells anabolism mostly affected the external regions of the disc, causing a loss up to 35% and 20% of the normal physiological content of GAGs at the CEP and AF, respectively (Fig 5). The detrimental effect of nicotine on disc cells anabolism is dose-dependent, and starts to manifest when the concentration of this molecule gets higher than 50 nM [20]. In the ‘light smoking’ scenario, nicotine levels were below this threshold value in most of the disc (Fig 4A), so that GAG concentration in the tissue did not significantly differ from that attained in a ‘non-smoking’ scenario. Similarly, for the ‘heavy smoking’ scenario, nicotine concentration was below 50 nM in most of NP, causing GAG levels in this disc region to remain almost unperturbed.

The reduced delivery of solutes to the IVD due to the constriction of the vascular network surrounding the tissue determined the level of malnutrition and impaired supply of anabolic agents (i.e., IGF-1) to disc cells. Accordingly, cell density and GAG content were reduced especially in the NP (Fig 6). These results are consistent with previous computational studies [33, 34, 36, 40, 41], and in agreement with the accepted notion that insufficient nutritional supply to IVD causes ECM degeneration [26, 28, 53–55].

The overall effect of tobacco-smoking was a depletion of GAG content across all disc regions (i.e., CEP, AF, and NP), and a reduction of cell density in both AF and NP. These results are consistent with a recent study from Wang and co-workers [19] in which 3 months-old mice were exposed to the equivalent of one pack of cigarette per day for 6 months (the equivalent of 15–20 years for humans). After treatment, murine discs exhibited reduced cell density and their proteoglycan content decreased up to 63% of the non-smoking control group. These data are in agreement with our model’s predictions (Fig 7C and 7D).

Previous numerical studies have shown that the IVD regions whose cell density is most affected by malnutrition are the inner NP, and the border between AF and NP [33, 36, 40, 41]. The results of this study show a different map of the areas of the IVD with low cell density, which is uniquely related to the effects of tobacco smoking (Fig 9). More specifically, because of the impaired nutritional supply due to vasoconstriction, cell densities of the innermost NP and AF are almost zero. Also, when ‘heavy smoking’ conditions are attained, the concentration of nicotine in the NP immediately close to CEP and adjacently to the outer AF is sufficiently high to down-regulate cell proliferation. In addition, the levels of nutrients and IGF-1 necessary for sustaining cellularity are low. This causes cell density to drop dramatically in those regions (Fig 9B). These results are consistent with histological evaluations of IVDs of both rat-smoking models and nicotine-injected rabbits, which exhibited signs of disc degeneration at the NP, and the inner and intermediate AF [15–18].

An association between quitting smoking and a mitigation of low back pain has been reported [56, 57]. However, this does not seem to be ascribed to a restoration of the nicotine-degenerated disc. In fact, the results of this analysis showed that the benefits to disc health associated with cessation of tobacco smoking were limited. At the CEP, GAG concentration fully recovered normal physiological values. In contrast, at the AF, cell density and GAG levels recovered up to 87% (light smoking) and 70% (heavy smoking) of the normal physiological values, and their corresponding values at NP were 74% (light smoking) and 55% (heavy smoking) (Fig 8). In agreement with our findings, histological evaluations in rat IVD after cessation of smoking revealed only partial recovery of the health of the disc tissue: although the overall amount of proteoglycans in the IVD tended to increase after cessation, no improvement was observed in those disc regions that are majorly affected by smoking-induced degeneration (e.g., intermediate AF) [18]. Since nicotine acts as an inflammatory agent for disc cells [17, 58], it may be plausible that the inflammatory cytokines produced upon smoking would migrate outside the disc and interact with nociceptors responsible for low back pain [59]. Hence, upon cessation of smoking, also the nicotine-mediated expression of inflammatory cytokines would stop, and low back pain could be attenuated despite the fact that the disc is still degenerated. These findings also suggest that, in the attempt to restore a degenerated IVD, therapeutic approaches additional to cessation of smoking may be pursued (i.e., growth factor therapy [60], cell-based therapy [61], etc. [62]). In the framework of this study, the effects of cell injection on cellularity and ECM composition in tobacco smoke degenerated discs were investigated. It was shown that, one year after injection, both cell density and GAG levels into the NP increased up to 95% (‘light smoking’ scenario) and 80% (‘heavy smoking’ scenario) of the values attained in normal physiological conditions (Fig 10). Such results suggest that, in theory, therapies based on cell injection may constitute a viable strategy for improving the homeostasis of a tobacco smoke degenerated IVD.

Some limitations of this analysis must be noted. The computational model used in this study did not include effects of physiological mechanical loading on the disc, which provides a direct contribution to the convective transport of solutes in the IVD [30, 38, 40, 63, 64], and it can also affect the metabolism of the cells in the tissue [65]. Also, another aspect the model did not account for is that the degeneration of the ECM due to tobacco smoking alters the morphology of the disc, and consequently, it also alters its transport properties. Accordingly, a reduction in GAG concentration determines a diminution of tissue swelling pressure and water-content [66], which causes a decrease in solute diffusivity [67, 68]. Hence, nutrients and IGF-1 delivery to disc cells may be lower than those computed in this analysis, so that GAG levels and cellularity may differ from those reported hereby. Moreover, in the IVD, several cell phenotypes (i.e. chondrocyte-like, fibroblast-like, notochordal, and articular chondrocytes [69]) are present in different proportions according to disc region, and their response to cigarette smoke may vary [70]. This model assumes that cell response to nicotine is the average response of all cell phenotypes present in murine NP [20]. Since the proportion of cell phenotypes in disc regions changes from animals to humans [71], the magnitude of the effects of exposing human IVD cells to nicotine may differ from what was simulated in this study. Furthermore, IGF-1 is not the sole up-regulator of disc cell anabolism, and several other agents have been shown to stimulate cell proliferation and extracellular matrix biosynthesis in IVD (e.g., TGF-β [72, 73], EGF [73], OP-1 [74], GDF-5 [75], BMP-2 [76], etc.). However, differently from IGF-1, only partial quantitative information is currently available on their effect on IVD anabolism. Hence, their contribution will be included in future studies, when more data will be available. Finally, in a recent preliminary study we have shown that nicotine-stimulated human NP cells express the inflammasome [58]. This is a multi-protein complex involved in the processing of the pro-inflammatory cytokines IL-1β and IL-18 [77], which are key mediators in the production of degradative enzymes of the ECM [78–80]. Due to the limited data currently available, these aspects were not considered in the present analysis, and will be included in a future study.

In conclusion, this study investigated the implications of two possible mechanisms for disc degeneration due to tobacco smoking: the nicotine-mediated down-regulation of cell proliferation and anabolism, and the reduced solute delivery to IVD due to vasoconstriction of the blood vessels surrounding the disc. Although presenting limitations, the model adopted in this study provided results whose validity is backed up by previously reported *in vivo* studies. More specifically, model’s predictions confirm the hypothesis that both mechanisms may play a significant role in IVD degeneration, acting on different disc regions at different extents: the nicotine down-regulating action mostly affects the GAG levels in the CEP, while vasoconstriction reduces GAGs and cell density in the inner disc. Also, the effectiveness of quitting smoking on the potential regeneration of a degenerated IVD was also investigated, and it was shown to have limited benefit on the health of the disc. These findings suggest that improvement in low back pain upon cessation of smoking cannot be attributed to a restoration of the integrity of the disc. In addition, these results indicate that, besides quitting smoking, additional treatments should be implemented in the attempt to recover the health of an IVD degenerated by tobacco smoking. For instance, cell injection into the disc, combined with smoke cessation, showed to significantly improve tissue health, suggesting that such approach might constitute a viable strategy for improving the homeostasis of a tobacco smoke-degenerated IVD. In this respect, the computational tool hereby presented could be useful for optimizing therapeutic protocols and designing *ad hoc* experimental validations.

## Appendix

The theoretical framework used in this study is a diffusive-reactive transport model describing the effects of IGF-1 and nicotine on ECM biosynthesis and cellularity in IVD. The IGF-mediated synthesis of ECM components and cell proliferation occur because of the interaction of the growth factor with its cell surface receptor. On the contrary, nicotine inhibits ECM production and cell proliferation in a dose-dependent fashion. Enhancement and inhibition of matrix biosynthesis and cell proliferation mediated by IGF-1 and nicotine affect the nutritional demand of disc cells. Accordingly, transport of nutrients and their metabolism are coupled with IGF-1 and nicotine transport and reaction kinetics. A brief model description is reported below.

### Transport of nutrients

The contribution of nutrients to disc homeostasis is taken into account by modeling transport of oxygen, glucose, and the metabolite lactate in the IVD. Mass balances for these species are:

$$\begin{array}{cccc} \frac{\partial c^{\alpha }}{\partial t}=D^{\alpha }\nabla^{2}c^{\alpha }+Q^{\alpha } & & & \left(\alpha =O_{2},Glucose,Lactate\right) \end{array},$$ (2)

where *D* ^*α*^ are solute diffusivities and *Q* ^*α*^ are the cellular metabolic rates of nutrients [30, 81]:

$$Q^{O_{2}}=-\frac{V{'}_{\max}\left(pH-4.95\right)c^{O_{2}}}{K{'}_{m}\left(pH-4.59\right)+c^{O_{2}}}\rho^{cell},$$ (3)

$$Q^{Lactate}=\exp\left(-2.47+0.93pH+0.16c^{O_{2}}-0.0058c^{O_{2}^{2}}\right)\frac{c^{Glucose}}{K_{m}+c^{Glucose}}\rho^{cell},$$ (4)

$$Q^{Glucose}=-0.5Q^{Lactate},$$ (5)

$$pH=-0.1c^{Lactate}+7.5,$$ (6)

where *V’*_*max*_ is the maximum consumption rate of oxygen, and *K’*_*m*_ and *K*_*m*_ are the Michaelis–Menten constants for oxygen and lactate, respectively [30].

### Transport of IGF-1

When trafficking in the ECM of IVD, IGF-1 competitively reacts with IGFBP and IGF-1R [82]. By interacting with the binding protein, a bound complex (IGF-1/IGFBP) forms. The IGF-1/IGFBP can reversibly dissociate into its constituents, or be degraded by IGFBP-protease to free IGF-1 [83]. Binding of IGF-1 to the cell surface receptor (IGF-1/IGF-1R) is also reversible and triggers both GAG production and cell proliferation. Moreover, formation of IGF-1/IGF-1R can be followed by a process of internalization of IGF-1, which eliminates the growth factor and frees the cell surface receptor [84]. Additional concurrent chemical reactions include both degradation of IGF-1 and enzymatic degradation of IGFBP via IGFBP-protease. Moreover, IGF-1 can be endogenously produced by disc cells. The following set of chemical reactions describes the interactions of IGF-1 with binding proteins and cell receptors [41, 85–88]:

$$IGF-1+IGFBP\begin{array}{c} \overset{k_{+1}}{\to }\\ \underset{k_{-1}}{\leftarrow } \end{array}IGF\text{-}1/IGFBP\overset{k_{BP}}{\to }IGF-1,$$ (7)

$$\begin{array}{c} IGF-1 \end{array}+IGF-1R\begin{array}{c} \overset{k_{+2}}{\to }\\ \underset{k_{-2}}{\leftarrow } \end{array}IGF-1/IGF-1R\overset{k_{i}}{\to }IGF-1R,$$ (8)

where, *k*_*+1*_ and *k*_*-1*_ are the binding association and dissociation rates between IGF-1 and IGFBP; *k*_*BP*_ is the degradation rate of binding proteins due to IGFBP-protease; *k*_*+2*_ and *k*_*-2*_ are the binding association and dissociation rates between IGF-1 and IGF-1R; *k*_*i*_ is the internalization rate of IGF-1 by disc cells. Accordingly, mass balance equations associated to the relations expressed in (4) are [41]:

$$\begin{array}{cccc} \frac{\partial c^{\beta }}{\partial t}=D^{\beta }\nabla^{2}c^{\beta }+Q^{\beta } & & & \left(\beta =IGF-1,IGFBP,IGF-1/IGFBP\right) \end{array},$$ (9)

$$\frac{dc^{IGF-1/IGF-1R}}{dt}=Q^{IGF-1/IGF-1R}.$$ (10)

In the above relations, *D* ^*β*^ are solute diffusivities, and *Q* ^*β*^ are source terms defined as [29, 41]:

$$\begin{array}{l} Q^{IGF-1}=k_{-1}c^{IGF-1/IGFBP}-k_{+1}c^{IGF-1}c^{IGFBP}+k_{BP}c^{IGF-1/IGFBP}\\ +k_{-2}c^{IGF-1/IGF-1R}-k_{+2}c^{IGF-1}\left(c^{RT}-c^{IGF-1/IGF-1R}\right)-k_{IGF}c^{IGF-1}+k_{IGF}^{endo}\rho^{cell}c^{IGF-1} \end{array}$$ (11)

$$Q^{IGFBP}=k_{-1}c^{IGF-1/IGFBP}-k_{+1}c^{IGF-1}c^{IGFBP}-k_{BP}c^{IGFBP},$$ (12)

$$Q^{IGF-1/IGFBP}=-k_{-1}c^{IGF-1/IGFBP}+k_{+1}c^{IGF-1}c^{IGFBP}-k_{BP}c^{IGF-1/IGFBP},$$ (13)

$$Q^{IGF-1/IGF-1R}=k_{+2}c^{I}\left(c^{RT}-c^{IGF-1/IGF-1R}\right)-k_{-2}c^{IGF-1/IGF-1R}-k_{i}c^{IGF-1/IGF-1R},$$ (14)

where *k*_*IGF*_ is the IGF-1 degradation rate; *c* ^*RT*^ is the total concentration of cell surface receptors (i.e., both free and IGF-bound), assumed to be linearly proportional to cell density [89]; *k*_*IGF*_^*endo*^ is the rate of endogenous production of IGF-1 and IGFBP per unit cell density, respectively; *ρ* ^*cell*^ is the cell density.

### Transport of nicotine

Upon tobacco smoking, nicotine is delivered to the IVD from the vascular network surrounding the tissue. Transport of this molecule in the disc is described by the following diffusive-reactive equation:

$$\frac{\partial c^{N}}{\partial t}=D^{N}\nabla^{2}c^{N}-k_{N}c^{N},$$ (15)

where *D* ^*Ν*^ is the diffusivity of nicotine in the IVD, and *k*_*N*_ its degradation rate.

### Viability, biosynthesis, and metabolism of IVD cells

Cell density in the IVD depends upon nutrients availability, and on agents stimulating or inhibiting cellular proliferation. *In vitro* studies reported that cell death is initiated when glucose concentration falls below 0.5mM, and at 0.2mM disc cells die in 3 days [26, 28, 42]. In addition, cell proliferation is enhanced by IGF-1 [43] and reduced by nicotine [20, 21]. Based on these experimental findings and on previous theoretical frameworks [40, 41, 90], the constitutive model for cell viability in IVD reads [41]:

$$\frac{\partial \rho^{cell}}{\partial t}=\alpha \cdot \left(\frac{c^{Glucose}-0.5-\left|c^{Glucose}-0.5\right|}{c^{Glucose}+0.2}\right)\rho^{cell}+\delta \cdot \alpha \cdot \left(R_{\rho }-1\right)\rho^{cell},$$ (16)

$$\delta =\{\begin{array}{c} 1\\ 0 \end{array}\begin{array}{c} \begin{array}{ccccc} & \text{for} & c^{Glucose}\geq 0.5\text{mM} & \text{and} & \rho^{cell}<\rho_{\max}^{cell} \end{array}\\ \begin{array}{ccccc} & \text{for} & c^{Glucose}<0.5\text{mM} & \text{or} & \rho^{cell}=\rho_{\max}^{cell} \end{array} \end{array},$$ (17)

where *α* is a time rate equal to 1 day^-1^ and *ρ*_max_^*cell*^ is the value of cell density in IVD in normal physiological conditions [91]. The parameter *R*_*ρ*_ is a cell proliferation rate coefficient defined as:

$$R_{\rho }=R_{\rho }^{IGF}\cdot R_{\rho }^{N},$$ (18)

where *R*_*ρ*_^*ΙGF*^ is a coefficient whose magnitude, included between 1 and 3.5, depends on the concentration of IGF-1 bound to cell receptors [41, 43]; *R*_*ρ*_^*Ν*^ is a coefficient whose magnitude, included between 0.27 and 1, depends on the concentration of nicotine in the disc [20] (Fig 3).

The rate of ECM biosynthesis also depends on both levels of IGF-1 [44] and nicotine [20, 21]. Such effects are accounted for into the model via the following relation [41]:

$$\frac{\partial c^{GAG}}{\partial t}=\gamma \cdot \alpha \cdot \left(R_{GAG}-1\right)c^{GAG},$$ (19)

$$\gamma =\{\begin{array}{c} 1\\ 0 \end{array}\begin{array}{c} \begin{array}{ccc} & \text{for} & c^{GAG}<c_{\max}^{GAG} \end{array}\\ \begin{array}{ccc} & \text{for} & c^{GAG}=c_{\max}^{GAG} \end{array} \end{array},$$ (20)

where *c* ^*GAG*^ is the GAG concentration and *c*_max_^*GAG*^ its level in IVD in normal physiological conditions [92]. The term *R*_*GAG*_ is a GAG synthesis rate coefficient defined as:

$$R_{GAG}=R_{GAG}^{IGF}\cdot R_{GAG}^{N},$$ (21)

where *R*_*GAG*_^*IGF*^ is a coefficient whose magnitude, included between 1 and 4.8, depends on the concentration of IGF-1 bound to cell receptors [41, 44]; *R*_*GAG*_^*N*^ is a coefficient whose magnitude, included between 0.64 and 1, depends on the concentration of nicotine in the disc [20] (Fig 3).

Since glycolysis is the main pathway of energy production, the IGF- and nicotine-mediated changes in ECM biosynthesis entail changes in glucose consumption and associated lactate production by disc cells [31]. Accordingly, the actual glucose and lactate metabolic rates are [41]:

$$Q^{Glucose^{*}}=R_{PG}Q^{Glucose},$$ (22)

$$Q^{Lactate^{*}}=R_{PG}Q^{Lactate}.$$ (23)

## Supporting Information

S1 File Nicotine-mediated down-regulation of cell proliferation and GAG biosynthesis in IVD. GAG concentration and cell density for both ‘light smoking’ and ‘heavy smoking’ scenarios are reported and compared to the corresponding values for ‘non-smoking’ scenario.
(PDF)

S2 File Reduction of solute supply to IVD due to tobacco smoking affects disc homeostasis. GAG concentration and cell density for both ‘light smoking’ and ‘heavy smoking’ scenarios are reported and compared to the corresponding values for ‘non-smoking’ scenario.
(PDF)

S3 File Effect of tobacco smoking on disc homeostasis. Glucose and oxygen levels, together with GAG concentration and cell density for both ‘light smoking’ and ‘heavy smoking’ scenarios are reported and compared to the corresponding values for ‘non-smoking’ scenario.
(PDF)

S4 File Partial recovery of IVD homeostasis at 1 year after smoke cessation. GAG levels and cell density in all disc regions are reported for both ‘light smoking’ and ‘heavy smoking’ scenarios. Data are normalized with respect to ‘non-smoking’ scenario.
(PDF)

S5 File Effect of cell injection on disc homeostasis. GAG levels and cell density in all disc regions are reported for both ‘light smoking’ and ‘heavy smoking’ scenarios. Data are normalized with respect to ‘non-smoking’ scenario.
(PDF)
