# Supplementary material for: Effects of Tobacco Smoking on the Degeneration of the Intervertebral Disc: A Finite Element Study
Source: PLoS One. 2015 Aug 24;10(8):e0136137. doi: 10.1371/journal.pone.0136137 (PMC4547737; doi:10.1371/journal.pone.0136137)
Supplement: S1 File — GAG concentration and cell density for both ‘light smoking’ and ‘heavy smoking’ scenarios are reported and compared to the corresponding values for ‘non-smoking’ scenario. (PDF) [file pone.0136137.s001.pdf]

	GAG [ug /mm^3]			Cell /mm^3		
**scenario**	**CEP**	**AF**	**NP**	**CEP**	**AF**	**NP**
non-smoker	81.81818	75.75758	105	15000	9000	4000
heavy smoker	53.77909	60.59091	96.999	15000	9000	4000
**ratio non-heavy**	65.73%	79.98%	92.38%	100.00%	100.00%	100.00%
light smoker	74.55273	74.34091	105	15000	9000	4000
**ratio non-light**	91.12%	98.13%	100.00%	100.00%	100.00%	100.00%
