# Supplementary material for: Effects of Tobacco Smoking on the Degeneration of the Intervertebral Disc: A Finite Element Study
Source: PLoS One. 2015 Aug 24;10(8):e0136137. doi: 10.1371/journal.pone.0136137 (PMC4547737; doi:10.1371/journal.pone.0136137)
Supplement: S2 File — GAG concentration and cell density for both ‘light smoking’ and ‘heavy smoking’ scenarios are reported and compared to the corresponding values for ‘non-smoking’ scenario. (PDF) [file pone.0136137.s002.pdf]

	GAG [ug /mm^3]			Cell /mm^3		
**scenario**	**CEP**	**AF**	**NP**	**CEP**	**AF**	**NP**
non-smoker	81.81818	75.75758	105	15000	9000	4000
heavy smoker	81.81818	59.72727	73.479	15000	7166.7	2848
**ratio non-heavy**	100.00%	78.84%	69.98%	100.00%	79.63%	71.20%
light smoker	81.81818	62.29545	74.991	15000	7360	2862
**ratio non-light**	100.00%	82.23%	71.42%	100.00%	81.78%	71.55%
