# Supplementary material for: Effects of Tobacco Smoking on the Degeneration of the Intervertebral Disc: A Finite Element Study
Source: PLoS One. 2015 Aug 24;10(8):e0136137. doi: 10.1371/journal.pone.0136137 (PMC4547737; doi:10.1371/journal.pone.0136137)
Supplement: S3 File — Glucose and oxygen levels, together with GAG concentration and cell density for both ‘light smoking’ and ‘heavy smoking’ scenarios are reported and compared to the corresponding values for ‘non-smoking’ scenario. (PDF) [file pone.0136137.s003.pdf]

	Glucose [mM]			Oxygen[kPa]		
**scenario**	**CEP**	**AF**	**NP**	**CEP**	**AF**	**NP**
non-smoker	2.75E+00	1.31E+00	8.01E-01	3.17E+00	1.93E+00	5.40E-01
heavy smoker	1.19E+00	7.40E-01	4.37E-01	7.79E-01	6.30E-01	2.54E-01
**ratio non-heavy**	43.26%	56.66%	54.63%	24.57%	32.64%	47.04%
light smoker	1.96E+00	9.66E-01	6.18E-01	1.47E+00	9.90E-01	3.09E-01
**ratio non-light**	71.41%	73.97%	77.26%	46.37%	51.30%	57.22%
	**GAG [ug /mm^3]**			**Cell /mm^3**		
**scenario**	**CEP**	**AF**	**NP**	**CEP**	**AF**	**NP**
non-smoker	81.81818	75.75758	105	15000	9000	4000
heavy smoker	31.01727	32.40909	69.0165	15000	5235.3	1730
**ratio non-heavy**	37.91%	42.78%	65.73%	100.00%	58.17%	43.25%
light smoker	59.01545	60.84091	95.676	15000	7397.1	2889.6
**ratio non-light**	72.13%	80.31%	91.12%	100.00%	82.19%	72.24%
