# Supplementary material for: Effects of Tobacco Smoking on the Degeneration of the Intervertebral Disc: A Finite Element Study
Source: PLoS One. 2015 Aug 24;10(8):e0136137. doi: 10.1371/journal.pone.0136137 (PMC4547737; doi:10.1371/journal.pone.0136137)
Supplement: S4 File — GAG levels and cell density in all disc regions are reported for both ‘light smoking’ and ‘heavy smoking’ scenarios. Data are normalized with respect to ‘non-smoking’ scenario. (PDF) [file pone.0136137.s004.pdf]

day	GAG-CEP	GAG-AF	GAG-NP	Cell-CEP	Cell-AF	Cell-NP
	Light Smoker					
**0**	93.7%	80.7%	72.1%	100.0%	82.2%	72.2%
**1**	100.0%	84.7%	72.1%	100.0%	84.2%	72.2%
**2**	100.0%	85.9%	72.2%	100.0%	85.4%	72.2%
**3**	100.0%	86.1%	72.2%	100.0%	85.6%	72.2%
**4**	100.0%	86.1%	72.3%	100.0%	85.6%	72.3%
**5**	100.0%	86.2%	72.5%	100.0%	85.7%	72.4%
**6**	100.0%	86.4%	72.7%	100.0%	85.8%	72.6%
**7**	100.0%	86.5%	72.9%	100.0%	86.0%	72.7%
**8**	100.0%	86.6%	73.0%	100.0%	86.0%	72.8%
**9**	100.0%	86.6%	73.1%	100.0%	86.1%	72.9%
**10**	100.0%	86.6%	73.2%	100.0%	86.1%	72.9%
**11**	100.0%	86.6%	73.2%	100.0%	86.1%	73.0%
**12**	100.0%	86.7%	73.3%	100.0%	86.1%	73.0%
**13**	100.0%	86.7%	73.3%	100.0%	86.2%	73.1%
**14**	100.0%	86.7%	73.3%	100.0%	86.2%	73.1%
**15**	100.0%	86.8%	73.4%	100.0%	86.2%	73.1%
**16**	100.0%	86.8%	73.4%	100.0%	86.2%	73.2%
**17**	100.0%	86.8%	73.4%	100.0%	86.2%	73.2%
**18**	100.0%	86.8%	73.5%	100.0%	86.2%	73.2%
**19**	100.0%	86.8%	73.5%	100.0%	86.3%	73.2%
**20**	100.0%	86.8%	73.5%	100.0%	86.3%	73.2%
**21**	100.0%	86.8%	73.5%	100.0%	86.3%	73.3%
**22**	100.0%	86.8%	73.5%	100.0%	86.3%	73.3%
**23**	100.0%	86.8%	73.6%	100.0%	86.3%	73.3%
**24**	100.0%	86.8%	73.6%	100.0%	86.3%	73.3%
**25**	100.0%	86.9%	73.6%	100.0%	86.3%	73.3%
**26**	100.0%	86.9%	73.6%	100.0%	86.3%	73.3%
**27**	100.0%	86.9%	73.6%	100.0%	86.3%	73.3%
**28**	100.0%	86.9%	73.6%	100.0%	86.3%	73.3%
**29**	100.0%	86.9%	73.6%	100.0%	86.3%	73.3%
**30**	100.0%	86.9%	73.6%	100.0%	86.3%	73.3%
**31**	100.0%	86.9%	73.6%	100.0%	86.3%	73.3%
**32**	100.0%	86.9%	73.6%	100.0%	86.3%	73.3%
**33**	100.0%	86.9%	73.6%	100.0%	86.3%	73.3%
**34**	100.0%	86.9%	73.6%	100.0%	86.3%	73.3%
**35**	100.0%	86.9%	73.6%	100.0%	86.3%	73.3%
**36**	100.0%	86.9%	73.6%	100.0%	86.3%	73.3%
**37**	100.0%	86.9%	73.6%	100.0%	86.3%	73.3%
**38**	100.0%	86.9%	73.6%	100.0%	86.3%	73.3%
**39**	100.0%	86.9%	73.6%	100.0%	86.4%	73.3%
**40**	100.0%	86.9%	73.6%	100.0%	86.4%	73.3%
**41**	100.0%	86.9%	73.6%	100.0%	86.4%	73.3%
**42**	100.0%	86.9%	73.6%	100.0%	86.4%	73.3%
**43**	100.0%	86.9%	73.6%	100.0%	86.4%	73.3%
**44**	100.0%	86.9%	73.6%	100.0%	86.4%	73.3%

**45**	100.0%	86.9%	73.6%	100.0%	86.4%	73.3%
**46**	100.0%	86.9%	73.6%	100.0%	86.4%	73.3%
**47**	100.0%	86.9%	73.6%	100.0%	86.4%	73.3%
**48**	100.0%	86.9%	73.6%	100.0%	86.4%	73.3%
**49**	100.0%	86.9%	73.6%	100.0%	86.4%	73.3%
**50**	100.0%	86.9%	73.6%	100.0%	86.4%	73.3%
**51**	100.0%	86.9%	73.6%	100.0%	86.4%	73.3%
**52**	100.0%	86.9%	73.6%	100.0%	86.4%	73.3%
**53**	100.0%	86.9%	73.6%	100.0%	86.4%	73.3%
**54**	100.0%	86.9%	73.6%	100.0%	86.4%	73.3%
**55**	100.0%	86.9%	73.6%	100.0%	86.4%	73.3%
**56**	100.0%	86.9%	73.6%	100.0%	86.4%	73.3%
**57**	100.0%	86.9%	73.6%	100.0%	86.4%	73.3%
**58**	100.0%	86.9%	73.6%	100.0%	86.4%	73.3%
**59**	100.0%	86.9%	73.6%	100.0%	86.4%	73.3%
**60**	100.0%	86.9%	73.6%	100.0%	86.4%	73.3%
**61**	100.0%	86.9%	73.6%	100.0%	86.4%	73.3%
**62**	100.0%	86.9%	73.6%	100.0%	86.4%	73.3%
**63**	100.0%	86.9%	73.6%	100.0%	86.4%	73.3%
**64**	100.0%	86.9%	73.6%	100.0%	86.4%	73.3%
**65**	100.0%	86.9%	73.6%	100.0%	86.4%	73.3%
**66**	100.0%	86.9%	73.6%	100.0%	86.4%	73.3%
**67**	100.0%	86.9%	73.6%	100.0%	86.4%	73.3%
**68**	100.0%	86.9%	73.6%	100.0%	86.4%	73.3%
**69**	100.0%	86.9%	73.6%	100.0%	86.4%	73.3%
**70**	100.0%	86.9%	73.6%	100.0%	86.4%	73.3%
**71**	100.0%	86.9%	73.6%	100.0%	86.4%	73.3%
**72**	100.0%	86.9%	73.6%	100.0%	86.4%	73.3%
**73**	100.0%	86.9%	73.6%	100.0%	86.4%	73.3%
**74**	100.0%	86.9%	73.6%	100.0%	86.4%	73.3%
**75**	100.0%	86.9%	73.6%	100.0%	86.4%	73.3%
**76**	100.0%	86.9%	73.6%	100.0%	86.4%	73.3%
**77**	100.0%	86.9%	73.6%	100.0%	86.4%	73.3%
**78**	100.0%	86.9%	73.6%	100.0%	86.4%	73.3%
**79**	100.0%	86.9%	73.6%	100.0%	86.4%	73.3%
**80**	100.0%	86.9%	73.6%	100.0%	86.4%	73.3%
**81**	100.0%	86.9%	73.6%	100.0%	86.4%	73.3%
**82**	100.0%	86.9%	73.6%	100.0%	86.4%	73.3%
**83**	100.0%	86.9%	73.6%	100.0%	86.4%	73.3%
**84**	100.0%	86.9%	73.6%	100.0%	86.4%	73.3%
**85**	100.0%	86.9%	73.6%	100.0%	86.4%	73.3%
**86**	100.0%	86.9%	73.6%	100.0%	86.4%	73.3%
**87**	100.0%	86.9%	73.6%	100.0%	86.4%	73.3%
**88**	100.0%	86.9%	73.6%	100.0%	86.4%	73.3%
**89**	100.0%	86.9%	73.6%	100.0%	86.4%	73.3%
**90**	100.0%	86.9%	73.6%	100.0%	86.4%	73.3%
**91**	100.0%	86.9%	73.6%	100.0%	86.4%	73.3%

**92**	100.0%	86.9%	73.6%	100.0%	86.4%	73.3%
**93**	100.0%	86.9%	73.6%	100.0%	86.4%	73.3%
**94**	100.0%	86.9%	73.6%	100.0%	86.4%	73.3%
**95**	100.0%	86.9%	73.6%	100.0%	86.4%	73.3%
**96**	100.0%	86.9%	73.6%	100.0%	86.4%	73.3%
**97**	100.0%	86.9%	73.6%	100.0%	86.4%	73.3%
**98**	100.0%	86.9%	73.6%	100.0%	86.4%	73.3%
**99**	100.0%	86.9%	73.6%	100.0%	86.4%	73.3%
**100**	100.0%	86.9%	73.6%	100.0%	86.4%	73.3%
**101**	100.0%	86.9%	73.6%	100.0%	86.4%	73.3%
**102**	100.0%	86.9%	73.6%	100.0%	86.4%	73.3%
**103**	100.0%	86.9%	73.6%	100.0%	86.4%	73.3%
**104**	100.0%	86.9%	73.6%	100.0%	86.4%	73.3%
**105**	100.0%	86.9%	73.6%	100.0%	86.4%	73.3%
**106**	100.0%	86.9%	73.6%	100.0%	86.4%	73.3%
**107**	100.0%	86.9%	73.6%	100.0%	86.4%	73.3%
**108**	100.0%	86.9%	73.6%	100.0%	86.4%	73.3%
**109**	100.0%	86.9%	73.6%	100.0%	86.4%	73.3%
**110**	100.0%	86.9%	73.6%	100.0%	86.4%	73.3%
**111**	100.0%	86.9%	73.6%	100.0%	86.4%	73.3%
**112**	100.0%	86.9%	73.6%	100.0%	86.4%	73.3%
**113**	100.0%	86.9%	73.6%	100.0%	86.4%	73.3%
**114**	100.0%	86.9%	73.6%	100.0%	86.4%	73.3%
**115**	100.0%	86.9%	73.6%	100.0%	86.4%	73.3%
**116**	100.0%	86.9%	73.6%	100.0%	86.4%	73.3%
**117**	100.0%	86.9%	73.6%	100.0%	86.4%	73.3%
**118**	100.0%	86.9%	73.6%	100.0%	86.4%	73.3%
**119**	100.0%	86.9%	73.6%	100.0%	86.4%	73.3%
**120**	100.0%	86.9%	73.6%	100.0%	86.4%	73.3%
**121**	100.0%	86.9%	73.6%	100.0%	86.4%	73.3%
**122**	100.0%	86.9%	73.6%	100.0%	86.4%	73.3%
**123**	100.0%	86.9%	73.6%	100.0%	86.4%	73.3%
**124**	100.0%	86.9%	73.6%	100.0%	86.4%	73.3%
**125**	100.0%	86.9%	73.6%	100.0%	86.4%	73.3%
**126**	100.0%	86.9%	73.6%	100.0%	86.4%	73.3%
**127**	100.0%	86.9%	73.6%	100.0%	86.4%	73.3%
**128**	100.0%	86.9%	73.6%	100.0%	86.4%	73.3%
**129**	100.0%	86.9%	73.6%	100.0%	86.4%	73.3%
**130**	100.0%	86.9%	73.6%	100.0%	86.4%	73.3%
**131**	100.0%	86.9%	73.6%	100.0%	86.4%	73.3%
**132**	100.0%	86.9%	73.6%	100.0%	86.4%	73.3%
**133**	100.0%	86.9%	73.6%	100.0%	86.4%	73.3%
**134**	100.0%	86.9%	73.6%	100.0%	86.4%	73.3%
**135**	100.0%	86.9%	73.6%	100.0%	86.4%	73.3%
**136**	100.0%	86.9%	73.6%	100.0%	86.4%	73.3%
**137**	100.0%	86.9%	73.6%	100.0%	86.4%	73.3%
**138**	100.0%	86.9%	73.6%	100.0%	86.4%	73.3%

**139**	100.0%	86.9%	73.6%	100.0%	86.4%	73.3%
**140**	100.0%	86.9%	73.6%	100.0%	86.4%	73.3%
**141**	100.0%	86.9%	73.6%	100.0%	86.4%	73.3%
**142**	100.0%	86.9%	73.6%	100.0%	86.4%	73.3%
**143**	100.0%	86.9%	73.6%	100.0%	86.4%	73.3%
**144**	100.0%	86.9%	73.6%	100.0%	86.4%	73.3%
**145**	100.0%	86.9%	73.6%	100.0%	86.4%	73.3%
**146**	100.0%	86.9%	73.6%	100.0%	86.4%	73.3%
**147**	100.0%	86.9%	73.6%	100.0%	86.4%	73.3%
**148**	100.0%	86.9%	73.6%	100.0%	86.4%	73.3%
**149**	100.0%	86.9%	73.6%	100.0%	86.4%	73.3%
**150**	100.0%	86.9%	73.6%	100.0%	86.4%	73.3%
**151**	100.0%	86.9%	73.6%	100.0%	86.4%	73.3%
**152**	100.0%	86.9%	73.6%	100.0%	86.4%	73.3%
**153**	100.0%	86.9%	73.6%	100.0%	86.4%	73.3%
**154**	100.0%	86.9%	73.6%	100.0%	86.4%	73.3%
**155**	100.0%	86.9%	73.6%	100.0%	86.4%	73.3%
**156**	100.0%	86.9%	73.6%	100.0%	86.4%	73.3%
**157**	100.0%	86.9%	73.6%	100.0%	86.4%	73.3%
**158**	100.0%	86.9%	73.6%	100.0%	86.4%	73.3%
**159**	100.0%	86.9%	73.6%	100.0%	86.4%	73.3%
**160**	100.0%	86.9%	73.6%	100.0%	86.4%	73.3%
**161**	100.0%	86.9%	73.6%	100.0%	86.4%	73.3%
**162**	100.0%	86.9%	73.6%	100.0%	86.4%	73.3%
**163**	100.0%	86.9%	73.6%	100.0%	86.4%	73.3%
**164**	100.0%	86.9%	73.6%	100.0%	86.4%	73.3%
**165**	100.0%	86.9%	73.6%	100.0%	86.4%	73.3%
**166**	100.0%	86.9%	73.6%	100.0%	86.4%	73.3%
**167**	100.0%	86.9%	73.6%	100.0%	86.4%	73.3%
**168**	100.0%	86.9%	73.6%	100.0%	86.4%	73.3%
**169**	100.0%	86.9%	73.6%	100.0%	86.4%	73.3%
**170**	100.0%	86.9%	73.6%	100.0%	86.4%	73.3%
**171**	100.0%	86.9%	73.6%	100.0%	86.4%	73.3%
**172**	100.0%	86.9%	73.6%	100.0%	86.4%	73.3%
**173**	100.0%	86.9%	73.6%	100.0%	86.4%	73.3%
**174**	100.0%	86.9%	73.6%	100.0%	86.4%	73.3%
**175**	100.0%	86.9%	73.6%	100.0%	86.4%	73.3%
**176**	100.0%	86.9%	73.6%	100.0%	86.4%	73.3%
**177**	100.0%	86.9%	73.6%	100.0%	86.4%	73.3%
**178**	100.0%	86.9%	73.6%	100.0%	86.4%	73.3%
**179**	100.0%	86.9%	73.6%	100.0%	86.4%	73.3%
**180**	100.0%	86.9%	73.6%	100.0%	86.4%	73.3%
**181**	100.0%	86.9%	73.6%	100.0%	86.4%	73.3%
**182**	100.0%	86.9%	73.6%	100.0%	86.4%	73.3%
**183**	100.0%	86.9%	73.6%	100.0%	86.4%	73.3%
**184**	100.0%	86.9%	73.6%	100.0%	86.4%	73.3%
**185**	100.0%	86.9%	73.6%	100.0%	86.4%	73.3%

**186**	100.0%	86.9%	73.6%	100.0%	86.4%	73.3%
**187**	100.0%	86.9%	73.6%	100.0%	86.4%	73.3%
**188**	100.0%	86.9%	73.6%	100.0%	86.4%	73.3%
**189**	100.0%	86.9%	73.6%	100.0%	86.4%	73.3%
**190**	100.0%	86.9%	73.6%	100.0%	86.4%	73.3%
**191**	100.0%	86.9%	73.6%	100.0%	86.4%	73.3%
**192**	100.0%	86.9%	73.6%	100.0%	86.4%	73.3%
**193**	100.0%	86.9%	73.6%	100.0%	86.4%	73.3%
**194**	100.0%	86.9%	73.6%	100.0%	86.4%	73.3%
**195**	100.0%	86.9%	73.6%	100.0%	86.4%	73.3%
**196**	100.0%	86.9%	73.6%	100.0%	86.4%	73.3%
**197**	100.0%	86.9%	73.6%	100.0%	86.4%	73.3%
**198**	100.0%	86.9%	73.6%	100.0%	86.4%	73.3%
**199**	100.0%	86.9%	73.6%	100.0%	86.4%	73.3%
**200**	100.0%	86.9%	73.6%	100.0%	86.4%	73.3%
**201**	100.0%	86.9%	73.6%	100.0%	86.4%	73.3%
**202**	100.0%	86.9%	73.6%	100.0%	86.4%	73.3%
**203**	100.0%	86.9%	73.6%	100.0%	86.4%	73.3%
**204**	100.0%	86.9%	73.6%	100.0%	86.4%	73.3%
**205**	100.0%	86.9%	73.6%	100.0%	86.4%	73.3%
**206**	100.0%	86.9%	73.6%	100.0%	86.4%	73.3%
**207**	100.0%	86.9%	73.6%	100.0%	86.4%	73.3%
**208**	100.0%	86.9%	73.6%	100.0%	86.4%	73.3%
**209**	100.0%	86.9%	73.6%	100.0%	86.4%	73.3%
**210**	100.0%	86.9%	73.6%	100.0%	86.4%	73.3%
**211**	100.0%	86.9%	73.6%	100.0%	86.4%	73.3%
**212**	100.0%	86.9%	73.6%	100.0%	86.4%	73.3%
**213**	100.0%	86.9%	73.6%	100.0%	86.4%	73.3%
**214**	100.0%	86.9%	73.6%	100.0%	86.4%	73.3%
**215**	100.0%	86.9%	73.6%	100.0%	86.4%	73.3%
**216**	100.0%	86.9%	73.6%	100.0%	86.4%	73.3%
**217**	100.0%	86.9%	73.6%	100.0%	86.4%	73.3%
**218**	100.0%	86.9%	73.6%	100.0%	86.4%	73.3%
**219**	100.0%	86.9%	73.6%	100.0%	86.4%	73.3%
**220**	100.0%	86.9%	73.6%	100.0%	86.4%	73.3%
**221**	100.0%	86.9%	73.6%	100.0%	86.4%	73.3%
**222**	100.0%	86.9%	73.6%	100.0%	86.4%	73.3%
**223**	100.0%	86.9%	73.6%	100.0%	86.4%	73.3%
**224**	100.0%	86.9%	73.6%	100.0%	86.4%	73.3%
**225**	100.0%	86.9%	73.6%	100.0%	86.4%	73.3%
**226**	100.0%	86.9%	73.6%	100.0%	86.4%	73.3%
**227**	100.0%	86.9%	73.6%	100.0%	86.4%	73.3%
**228**	100.0%	86.9%	73.6%	100.0%	86.4%	73.3%
**229**	100.0%	86.9%	73.6%	100.0%	86.4%	73.3%
**230**	100.0%	86.9%	73.6%	100.0%	86.4%	73.3%
**231**	100.0%	86.9%	73.6%	100.0%	86.4%	73.3%
**232**	100.0%	86.9%	73.6%	100.0%	86.4%	73.3%

**233**	100.0%	86.9%	73.6%	100.0%	86.4%	73.3%
**234**	100.0%	86.9%	73.6%	100.0%	86.4%	73.3%
**235**	100.0%	86.9%	73.6%	100.0%	86.4%	73.3%
**236**	100.0%	86.9%	73.6%	100.0%	86.4%	73.3%
**237**	100.0%	86.9%	73.6%	100.0%	86.4%	73.3%
**238**	100.0%	86.9%	73.6%	100.0%	86.4%	73.3%
**239**	100.0%	86.9%	73.6%	100.0%	86.4%	73.3%
**240**	100.0%	86.9%	73.6%	100.0%	86.4%	73.3%
**241**	100.0%	86.9%	73.6%	100.0%	86.4%	73.3%
**242**	100.0%	86.9%	73.6%	100.0%	86.4%	73.3%
**243**	100.0%	86.9%	73.6%	100.0%	86.4%	73.3%
**244**	100.0%	86.9%	73.6%	100.0%	86.4%	73.3%
**245**	100.0%	86.9%	73.6%	100.0%	86.4%	73.3%
**246**	100.0%	86.9%	73.6%	100.0%	86.4%	73.3%
**247**	100.0%	86.9%	73.6%	100.0%	86.4%	73.3%
**248**	100.0%	86.9%	73.6%	100.0%	86.4%	73.3%
**249**	100.0%	86.9%	73.6%	100.0%	86.4%	73.3%
**250**	100.0%	86.9%	73.6%	100.0%	86.4%	73.3%
**251**	100.0%	86.9%	73.6%	100.0%	86.4%	73.3%
**252**	100.0%	86.9%	73.6%	100.0%	86.4%	73.3%
**253**	100.0%	86.9%	73.6%	100.0%	86.4%	73.3%
**254**	100.0%	86.9%	73.6%	100.0%	86.4%	73.3%
**255**	100.0%	86.9%	73.6%	100.0%	86.4%	73.3%
**256**	100.0%	86.9%	73.6%	100.0%	86.4%	73.3%
**257**	100.0%	86.9%	73.6%	100.0%	86.4%	73.3%
**258**	100.0%	86.9%	73.6%	100.0%	86.4%	73.3%
**259**	100.0%	86.9%	73.6%	100.0%	86.4%	73.3%
**260**	100.0%	86.9%	73.6%	100.0%	86.4%	73.3%
**261**	100.0%	86.9%	73.6%	100.0%	86.4%	73.3%
**262**	100.0%	86.9%	73.6%	100.0%	86.4%	73.3%
**263**	100.0%	86.9%	73.6%	100.0%	86.4%	73.3%
**264**	100.0%	86.9%	73.6%	100.0%	86.4%	73.3%
**265**	100.0%	86.9%	73.6%	100.0%	86.4%	73.3%
**266**	100.0%	86.9%	73.6%	100.0%	86.4%	73.3%
**267**	100.0%	86.9%	73.6%	100.0%	86.4%	73.3%
**268**	100.0%	86.9%	73.6%	100.0%	86.4%	73.3%
**269**	100.0%	86.9%	73.6%	100.0%	86.4%	73.3%
**270**	100.0%	86.9%	73.6%	100.0%	86.4%	73.3%
**271**	100.0%	86.9%	73.6%	100.0%	86.4%	73.3%
**272**	100.0%	86.9%	73.6%	100.0%	86.4%	73.3%
**273**	100.0%	86.9%	73.6%	100.0%	86.4%	73.3%
**274**	100.0%	86.9%	73.6%	100.0%	86.4%	73.3%
**275**	100.0%	86.9%	73.6%	100.0%	86.4%	73.3%
**276**	100.0%	86.9%	73.6%	100.0%	86.4%	73.3%
**277**	100.0%	86.9%	73.6%	100.0%	86.4%	73.3%
**278**	100.0%	86.9%	73.6%	100.0%	86.4%	73.3%
**279**	100.0%	86.9%	73.6%	100.0%	86.4%	73.3%

**280**	100.0%	86.9%	73.6%	100.0%	86.4%	73.3%
**281**	100.0%	86.9%	73.6%	100.0%	86.4%	73.3%
**282**	100.0%	86.9%	73.6%	100.0%	86.4%	73.3%
**283**	100.0%	86.9%	73.6%	100.0%	86.4%	73.3%
**284**	100.0%	86.9%	73.6%	100.0%	86.4%	73.3%
**285**	100.0%	86.9%	73.6%	100.0%	86.4%	73.3%
**286**	100.0%	86.9%	73.6%	100.0%	86.4%	73.3%
**287**	100.0%	86.9%	73.6%	100.0%	86.4%	73.3%
**288**	100.0%	86.9%	73.6%	100.0%	86.4%	73.3%
**289**	100.0%	86.9%	73.6%	100.0%	86.4%	73.3%
**290**	100.0%	86.9%	73.6%	100.0%	86.4%	73.3%
**291**	100.0%	86.9%	73.6%	100.0%	86.4%	73.3%
**292**	100.0%	86.9%	73.6%	100.0%	86.4%	73.3%
**293**	100.0%	86.9%	73.6%	100.0%	86.4%	73.3%
**294**	100.0%	86.9%	73.6%	100.0%	86.4%	73.3%
**295**	100.0%	86.9%	73.6%	100.0%	86.4%	73.3%
**296**	100.0%	86.9%	73.6%	100.0%	86.4%	73.3%
**297**	100.0%	86.9%	73.6%	100.0%	86.4%	73.3%
**298**	100.0%	86.9%	73.6%	100.0%	86.4%	73.3%
**299**	100.0%	86.9%	73.6%	100.0%	86.4%	73.3%
**300**	100.0%	86.9%	73.6%	100.0%	86.4%	73.3%
**301**	100.0%	86.9%	73.6%	100.0%	86.4%	73.3%
**302**	100.0%	86.9%	73.6%	100.0%	86.4%	73.3%
**303**	100.0%	86.9%	73.6%	100.0%	86.4%	73.3%
**304**	100.0%	86.9%	73.6%	100.0%	86.4%	73.3%
**305**	100.0%	86.9%	73.6%	100.0%	86.4%	73.3%
**306**	100.0%	86.9%	73.6%	100.0%	86.4%	73.3%
**307**	100.0%	86.9%	73.6%	100.0%	86.4%	73.3%
**308**	100.0%	86.9%	73.6%	100.0%	86.4%	73.3%
**309**	100.0%	86.9%	73.6%	100.0%	86.4%	73.3%
**310**	100.0%	86.9%	73.6%	100.0%	86.4%	73.3%
**311**	100.0%	86.9%	73.6%	100.0%	86.4%	73.3%
**312**	100.0%	86.9%	73.6%	100.0%	86.4%	73.3%
**313**	100.0%	86.9%	73.6%	100.0%	86.4%	73.3%
**314**	100.0%	86.9%	73.6%	100.0%	86.4%	73.3%
**315**	100.0%	86.9%	73.6%	100.0%	86.4%	73.3%
**316**	100.0%	86.9%	73.6%	100.0%	86.4%	73.3%
**317**	100.0%	86.9%	73.6%	100.0%	86.4%	73.3%
**318**	100.0%	86.9%	73.6%	100.0%	86.4%	73.3%
**319**	100.0%	86.9%	73.6%	100.0%	86.4%	73.3%
**320**	100.0%	86.9%	73.6%	100.0%	86.4%	73.3%
**321**	100.0%	86.9%	73.6%	100.0%	86.4%	73.3%
**322**	100.0%	86.9%	73.6%	100.0%	86.4%	73.3%
**323**	100.0%	86.9%	73.6%	100.0%	86.4%	73.3%
**324**	100.0%	86.9%	73.6%	100.0%	86.4%	73.3%
**325**	100.0%	86.9%	73.6%	100.0%	86.4%	73.3%
**326**	100.0%	86.9%	73.6%	100.0%	86.4%	73.3%

**327**	100.0%	86.9%	73.6%	100.0%	86.4%	73.3%
**328**	100.0%	86.9%	73.6%	100.0%	86.4%	73.3%
**329**	100.0%	86.9%	73.6%	100.0%	86.4%	73.3%
**330**	100.0%	86.9%	73.6%	100.0%	86.4%	73.3%
**331**	100.0%	86.9%	73.6%	100.0%	86.4%	73.3%
**332**	100.0%	86.9%	73.6%	100.0%	86.4%	73.3%
**333**	100.0%	86.9%	73.6%	100.0%	86.4%	73.3%
**334**	100.0%	86.9%	73.6%	100.0%	86.4%	73.3%
**335**	100.0%	86.9%	73.6%	100.0%	86.4%	73.3%
**336**	100.0%	86.9%	73.6%	100.0%	86.4%	73.3%
**337**	100.0%	86.9%	73.6%	100.0%	86.4%	73.3%
**338**	100.0%	86.9%	73.6%	100.0%	86.4%	73.3%
**339**	100.0%	86.9%	73.6%	100.0%	86.4%	73.3%
**340**	100.0%	86.9%	73.6%	100.0%	86.4%	73.3%
**341**	100.0%	86.9%	73.6%	100.0%	86.4%	73.3%
**342**	100.0%	86.9%	73.6%	100.0%	86.4%	73.3%
**343**	100.0%	86.9%	73.6%	100.0%	86.4%	73.3%
**344**	100.0%	86.9%	73.6%	100.0%	86.4%	73.3%
**345**	100.0%	86.9%	73.6%	100.0%	86.4%	73.3%
**346**	100.0%	86.9%	73.6%	100.0%	86.4%	73.3%
**347**	100.0%	86.9%	73.6%	100.0%	86.4%	73.3%
**348**	100.0%	86.9%	73.6%	100.0%	86.4%	73.3%
**349**	100.0%	86.9%	73.6%	100.0%	86.4%	73.3%
**350**	100.0%	86.9%	73.6%	100.0%	86.4%	73.3%
**351**	100.0%	86.9%	73.6%	100.0%	86.4%	73.3%
**352**	100.0%	86.9%	73.6%	100.0%	86.4%	73.3%
**353**	100.0%	86.9%	73.6%	100.0%	86.4%	73.3%
**354**	100.0%	86.9%	73.6%	100.0%	86.4%	73.3%
**355**	100.0%	86.9%	73.6%	100.0%	86.4%	73.3%
**356**	100.0%	86.9%	73.6%	100.0%	86.4%	73.3%
**357**	100.0%	86.9%	73.6%	100.0%	86.4%	73.3%
**358**	100.0%	86.9%	73.6%	100.0%	86.4%	73.3%
**359**	100.0%	86.9%	73.6%	100.0%	86.4%	73.3%
**360**	100.0%	86.9%	73.6%	100.0%	86.4%	73.3%
**361**	100.0%	86.9%	73.6%	100.0%	86.4%	73.3%
**362**	100.0%	86.9%	73.6%	100.0%	86.4%	73.3%
**363**	100.0%	86.9%	73.6%	100.0%	86.4%	73.3%
**364**	100.0%	86.9%	73.6%	100.0%	86.4%	73.3%

	Heavy Smoker					
**day**	GAG-CEP	GAG-AF	GAG-NP	Cell-CEP	Cell-AF	Cell-NP
**0**	69.4%	43.2%	37.9%	100.0%	58.2%	43.3%
**1**	100.0%	65.9%	44.2%	100.0%	65.8%	45.8%
**2**	100.0%	67.2%	45.0%	100.0%	67.1%	46.4%
**3**	100.0%	67.6%	46.0%	100.0%	67.5%	47.1%
**4**	100.0%	67.8%	47.1%	100.0%	67.7%	48.1%
**5**	100.0%	68.2%	48.1%	100.0%	68.1%	48.9%

**6**	100.0%	68.5%	49.0%	100.0%	68.3%	49.8%
**7**	100.0%	68.5%	49.8%	100.0%	68.4%	50.5%
**8**	100.0%	68.6%	50.6%	100.0%	68.5%	51.2%
**9**	100.0%	68.8%	51.1%	100.0%	68.7%	51.7%
**10**	100.0%	69.0%	51.6%	100.0%	68.9%	52.1%
**11**	100.0%	69.1%	51.9%	100.0%	69.0%	52.4%
**12**	100.0%	69.1%	52.2%	100.0%	69.0%	52.7%
**13**	100.0%	69.1%	52.4%	100.0%	69.0%	52.9%
**14**	100.0%	69.2%	52.6%	100.0%	69.1%	53.1%
**15**	100.0%	69.2%	52.7%	100.0%	69.1%	53.2%
**16**	100.0%	69.2%	52.9%	100.0%	69.1%	53.4%
**17**	100.0%	69.2%	53.0%	100.0%	69.1%	53.5%
**18**	100.0%	69.2%	53.1%	100.0%	69.1%	53.6%
**19**	100.0%	69.2%	53.2%	100.0%	69.1%	53.7%
**20**	100.0%	69.2%	53.3%	100.0%	69.1%	53.8%
**21**	100.0%	69.2%	53.5%	100.0%	69.1%	53.9%
**22**	100.0%	69.2%	53.5%	100.0%	69.1%	54.0%
**23**	100.0%	69.2%	53.6%	100.0%	69.1%	54.0%
**24**	100.0%	69.2%	53.6%	100.0%	69.1%	54.1%
**25**	100.0%	69.2%	53.6%	100.0%	69.1%	54.1%
**26**	100.0%	69.2%	53.7%	100.0%	69.1%	54.1%
**27**	100.0%	69.2%	53.7%	100.0%	69.1%	54.1%
**28**	100.0%	69.3%	53.7%	100.0%	69.1%	54.2%
**29**	100.0%	69.3%	53.7%	100.0%	69.2%	54.2%
**30**	100.0%	69.3%	53.7%	100.0%	69.2%	54.2%
**31**	100.0%	69.3%	53.7%	100.0%	69.2%	54.2%
**32**	100.0%	69.3%	53.8%	100.0%	69.2%	54.2%
**33**	100.0%	69.3%	53.8%	100.0%	69.2%	54.2%
**34**	100.0%	69.3%	53.8%	100.0%	69.2%	54.2%
**35**	100.0%	69.3%	53.8%	100.0%	69.2%	54.2%
**36**	100.0%	69.3%	53.8%	100.0%	69.2%	54.2%
**37**	100.0%	69.3%	53.8%	100.0%	69.2%	54.2%
**38**	100.0%	69.3%	53.8%	100.0%	69.2%	54.2%
**39**	100.0%	69.3%	53.8%	100.0%	69.2%	54.2%
**40**	100.0%	69.3%	53.8%	100.0%	69.2%	54.2%
**41**	100.0%	69.3%	53.8%	100.0%	69.2%	54.2%
**42**	100.0%	69.3%	53.8%	100.0%	69.2%	54.2%
**43**	100.0%	69.3%	53.8%	100.0%	69.2%	54.2%
**44**	100.0%	69.3%	53.8%	100.0%	69.2%	54.2%
**45**	100.0%	69.3%	53.8%	100.0%	69.2%	54.2%
**46**	100.0%	69.3%	53.8%	100.0%	69.2%	54.2%
**47**	100.0%	69.3%	53.8%	100.0%	69.2%	54.2%
**48**	100.0%	69.3%	53.8%	100.0%	69.2%	54.2%
**49**	100.0%	69.3%	53.8%	100.0%	69.2%	54.2%
**50**	100.0%	69.3%	53.8%	100.0%	69.2%	54.2%
**51**	100.0%	69.3%	53.8%	100.0%	69.2%	54.2%
**52**	100.0%	69.3%	53.8%	100.0%	69.2%	54.2%

**53**	100.0%	69.3%	53.8%	100.0%	69.2%	54.2%
**54**	100.0%	69.3%	53.8%	100.0%	69.2%	54.2%
**55**	100.0%	69.3%	53.8%	100.0%	69.2%	54.2%
**56**	100.0%	69.3%	53.8%	100.0%	69.2%	54.2%
**57**	100.0%	69.3%	53.8%	100.0%	69.2%	54.2%
**58**	100.0%	69.3%	53.8%	100.0%	69.2%	54.2%
**59**	100.0%	69.3%	53.8%	100.0%	69.2%	54.2%
**60**	100.0%	69.3%	53.8%	100.0%	69.2%	54.2%
**61**	100.0%	69.3%	53.8%	100.0%	69.2%	54.2%
**62**	100.0%	69.3%	53.8%	100.0%	69.2%	54.2%
**63**	100.0%	69.3%	53.8%	100.0%	69.2%	54.2%
**64**	100.0%	69.3%	53.8%	100.0%	69.2%	54.2%
**65**	100.0%	69.3%	53.8%	100.0%	69.2%	54.2%
**66**	100.0%	69.3%	53.8%	100.0%	69.2%	54.2%
**67**	100.0%	69.3%	53.8%	100.0%	69.2%	54.2%
**68**	100.0%	69.3%	53.8%	100.0%	69.2%	54.2%
**69**	100.0%	69.3%	53.8%	100.0%	69.2%	54.2%
**70**	100.0%	69.3%	53.8%	100.0%	69.2%	54.2%
**71**	100.0%	69.3%	53.8%	100.0%	69.2%	54.2%
**72**	100.0%	69.3%	53.8%	100.0%	69.2%	54.2%
**73**	100.0%	69.3%	53.8%	100.0%	69.2%	54.2%
**74**	100.0%	69.3%	53.8%	100.0%	69.2%	54.2%
**75**	100.0%	69.3%	53.8%	100.0%	69.2%	54.2%
**76**	100.0%	69.3%	53.8%	100.0%	69.2%	54.2%
**77**	100.0%	69.3%	53.8%	100.0%	69.2%	54.2%
**78**	100.0%	69.3%	53.8%	100.0%	69.2%	54.2%
**79**	100.0%	69.3%	53.8%	100.0%	69.2%	54.2%
**80**	100.0%	69.3%	53.8%	100.0%	69.2%	54.2%
**81**	100.0%	69.3%	53.8%	100.0%	69.2%	54.2%
**82**	100.0%	69.3%	53.8%	100.0%	69.2%	54.2%
**83**	100.0%	69.3%	53.8%	100.0%	69.2%	54.2%
**84**	100.0%	69.3%	53.8%	100.0%	69.2%	54.2%
**85**	100.0%	69.3%	53.8%	100.0%	69.2%	54.2%
**86**	100.0%	69.3%	53.8%	100.0%	69.2%	54.2%
**87**	100.0%	69.3%	53.8%	100.0%	69.2%	54.2%
**88**	100.0%	69.3%	53.8%	100.0%	69.2%	54.2%
**89**	100.0%	69.3%	53.8%	100.0%	69.2%	54.2%
**90**	100.0%	69.3%	53.8%	100.0%	69.2%	54.2%
**91**	100.0%	69.3%	53.8%	100.0%	69.2%	54.2%
**92**	100.0%	69.3%	53.8%	100.0%	69.2%	54.2%
**93**	100.0%	69.3%	53.8%	100.0%	69.2%	54.2%
**94**	100.0%	69.3%	53.8%	100.0%	69.2%	54.2%
**95**	100.0%	69.3%	53.8%	100.0%	69.2%	54.2%
**96**	100.0%	69.3%	53.8%	100.0%	69.2%	54.2%
**97**	100.0%	69.3%	53.8%	100.0%	69.2%	54.2%
**98**	100.0%	69.3%	53.8%	100.0%	69.2%	54.2%
**99**	100.0%	69.3%	53.8%	100.0%	69.2%	54.2%

**100**	100.0%	69.3%	53.8%	100.0%	69.2%	54.2%
**101**	100.0%	69.3%	53.8%	100.0%	69.2%	54.2%
**102**	100.0%	69.3%	53.8%	100.0%	69.2%	54.2%
**103**	100.0%	69.3%	53.8%	100.0%	69.2%	54.2%
**104**	100.0%	69.3%	53.8%	100.0%	69.2%	54.2%
**105**	100.0%	69.3%	53.8%	100.0%	69.2%	54.2%
**106**	100.0%	69.3%	53.8%	100.0%	69.2%	54.2%
**107**	100.0%	69.3%	53.8%	100.0%	69.2%	54.2%
**108**	100.0%	69.3%	53.8%	100.0%	69.2%	54.2%
**109**	100.0%	69.3%	53.8%	100.0%	69.2%	54.2%
**110**	100.0%	69.3%	53.8%	100.0%	69.2%	54.2%
**111**	100.0%	69.3%	53.8%	100.0%	69.2%	54.2%
**112**	100.0%	69.3%	53.8%	100.0%	69.2%	54.2%
**113**	100.0%	69.3%	53.8%	100.0%	69.2%	54.2%
**114**	100.0%	69.3%	53.8%	100.0%	69.2%	54.2%
**115**	100.0%	69.3%	53.8%	100.0%	69.2%	54.2%
**116**	100.0%	69.3%	53.8%	100.0%	69.2%	54.2%
**117**	100.0%	69.3%	53.8%	100.0%	69.2%	54.2%
**118**	100.0%	69.3%	53.8%	100.0%	69.2%	54.2%
**119**	100.0%	69.3%	53.8%	100.0%	69.2%	54.2%
**120**	100.0%	69.3%	53.8%	100.0%	69.2%	54.2%
**121**	100.0%	69.3%	53.8%	100.0%	69.2%	54.2%
**122**	100.0%	69.3%	53.8%	100.0%	69.2%	54.2%
**123**	100.0%	69.3%	53.8%	100.0%	69.2%	54.2%
**124**	100.0%	69.3%	53.8%	100.0%	69.2%	54.2%
**125**	100.0%	69.3%	53.8%	100.0%	69.2%	54.2%
**126**	100.0%	69.3%	53.8%	100.0%	69.2%	54.2%
**127**	100.0%	69.3%	53.8%	100.0%	69.2%	54.2%
**128**	100.0%	69.3%	53.8%	100.0%	69.2%	54.2%
**129**	100.0%	69.3%	53.8%	100.0%	69.2%	54.2%
**130**	100.0%	69.3%	53.8%	100.0%	69.2%	54.2%
**131**	100.0%	69.3%	53.8%	100.0%	69.2%	54.2%
**132**	100.0%	69.3%	53.8%	100.0%	69.2%	54.2%
**133**	100.0%	69.3%	53.8%	100.0%	69.2%	54.2%
**134**	100.0%	69.3%	53.8%	100.0%	69.2%	54.2%
**135**	100.0%	69.3%	53.8%	100.0%	69.2%	54.2%
**136**	100.0%	69.3%	53.8%	100.0%	69.2%	54.2%
**137**	100.0%	69.3%	53.8%	100.0%	69.2%	54.2%
**138**	100.0%	69.3%	53.8%	100.0%	69.2%	54.2%
**139**	100.0%	69.3%	53.8%	100.0%	69.2%	54.2%
**140**	100.0%	69.3%	53.8%	100.0%	69.2%	54.2%
**141**	100.0%	69.3%	53.8%	100.0%	69.2%	54.2%
**142**	100.0%	69.3%	53.8%	100.0%	69.2%	54.2%
**143**	100.0%	69.3%	53.8%	100.0%	69.2%	54.2%
**144**	100.0%	69.3%	53.8%	100.0%	69.2%	54.2%
**145**	100.0%	69.3%	53.8%	100.0%	69.2%	54.2%
**146**	100.0%	69.3%	53.8%	100.0%	69.2%	54.2%

**147**	100.0%	69.3%	53.8%	100.0%	69.2%	54.2%
**148**	100.0%	69.3%	53.8%	100.0%	69.2%	54.2%
**149**	100.0%	69.3%	53.8%	100.0%	69.2%	54.2%
**150**	100.0%	69.3%	53.8%	100.0%	69.2%	54.2%
**151**	100.0%	69.3%	53.8%	100.0%	69.2%	54.2%
**152**	100.0%	69.3%	53.8%	100.0%	69.2%	54.2%
**153**	100.0%	69.3%	53.8%	100.0%	69.2%	54.2%
**154**	100.0%	69.3%	53.8%	100.0%	69.2%	54.2%
**155**	100.0%	69.3%	53.8%	100.0%	69.2%	54.2%
**156**	100.0%	69.3%	53.8%	100.0%	69.2%	54.2%
**157**	100.0%	69.3%	53.8%	100.0%	69.2%	54.2%
**158**	100.0%	69.3%	53.8%	100.0%	69.2%	54.2%
**159**	100.0%	69.3%	53.8%	100.0%	69.2%	54.2%
**160**	100.0%	69.3%	53.8%	100.0%	69.2%	54.2%
**161**	100.0%	69.3%	53.8%	100.0%	69.2%	54.2%
**162**	100.0%	69.3%	53.8%	100.0%	69.2%	54.2%
**163**	100.0%	69.3%	53.8%	100.0%	69.2%	54.2%
**164**	100.0%	69.3%	53.8%	100.0%	69.2%	54.2%
**165**	100.0%	69.3%	53.8%	100.0%	69.2%	54.2%
**166**	100.0%	69.3%	53.8%	100.0%	69.2%	54.2%
**167**	100.0%	69.3%	53.8%	100.0%	69.2%	54.2%
**168**	100.0%	69.3%	53.8%	100.0%	69.2%	54.2%
**169**	100.0%	69.3%	53.8%	100.0%	69.2%	54.2%
**170**	100.0%	69.3%	53.8%	100.0%	69.2%	54.2%
**171**	100.0%	69.3%	53.8%	100.0%	69.2%	54.2%
**172**	100.0%	69.3%	53.8%	100.0%	69.2%	54.2%
**173**	100.0%	69.3%	53.8%	100.0%	69.2%	54.2%
**174**	100.0%	69.3%	53.8%	100.0%	69.2%	54.2%
**175**	100.0%	69.3%	53.8%	100.0%	69.2%	54.2%
**176**	100.0%	69.3%	53.8%	100.0%	69.2%	54.2%
**177**	100.0%	69.3%	53.8%	100.0%	69.2%	54.2%
**178**	100.0%	69.3%	53.8%	100.0%	69.2%	54.2%
**179**	100.0%	69.3%	53.8%	100.0%	69.2%	54.2%
**180**	100.0%	69.3%	53.8%	100.0%	69.2%	54.2%
**181**	100.0%	69.3%	53.8%	100.0%	69.2%	54.2%
**182**	100.0%	69.3%	53.8%	100.0%	69.2%	54.2%
**183**	100.0%	69.3%	53.8%	100.0%	69.2%	54.2%
**184**	100.0%	69.3%	53.8%	100.0%	69.2%	54.2%
**185**	100.0%	69.3%	53.8%	100.0%	69.2%	54.2%
**186**	100.0%	69.3%	53.8%	100.0%	69.2%	54.2%
**187**	100.0%	69.3%	53.8%	100.0%	69.2%	54.2%
**188**	100.0%	69.3%	53.8%	100.0%	69.2%	54.2%
**189**	100.0%	69.3%	53.8%	100.0%	69.2%	54.2%
**190**	100.0%	69.3%	53.8%	100.0%	69.2%	54.2%
**191**	100.0%	69.3%	53.8%	100.0%	69.2%	54.2%
**192**	100.0%	69.3%	53.8%	100.0%	69.2%	54.2%
**193**	100.0%	69.3%	53.8%	100.0%	69.2%	54.2%

**194**	100.0%	69.3%	53.8%	100.0%	69.2%	54.2%
**195**	100.0%	69.3%	53.8%	100.0%	69.2%	54.2%
**196**	100.0%	69.3%	53.8%	100.0%	69.2%	54.2%
**197**	100.0%	69.3%	53.8%	100.0%	69.2%	54.2%
**198**	100.0%	69.3%	53.8%	100.0%	69.2%	54.2%
**199**	100.0%	69.3%	53.8%	100.0%	69.2%	54.2%
**200**	100.0%	69.3%	53.8%	100.0%	69.2%	54.2%
**201**	100.0%	69.3%	53.8%	100.0%	69.2%	54.2%
**202**	100.0%	69.3%	53.8%	100.0%	69.2%	54.2%
**203**	100.0%	69.3%	53.8%	100.0%	69.2%	54.2%
**204**	100.0%	69.3%	53.8%	100.0%	69.2%	54.2%
**205**	100.0%	69.3%	53.8%	100.0%	69.2%	54.2%
**206**	100.0%	69.3%	53.8%	100.0%	69.2%	54.2%
**207**	100.0%	69.3%	53.8%	100.0%	69.2%	54.2%
**208**	100.0%	69.3%	53.8%	100.0%	69.2%	54.2%
**209**	100.0%	69.3%	53.8%	100.0%	69.2%	54.2%
**210**	100.0%	69.3%	53.8%	100.0%	69.2%	54.2%
**211**	100.0%	69.3%	53.8%	100.0%	69.2%	54.2%
**212**	100.0%	69.3%	53.8%	100.0%	69.2%	54.2%
**213**	100.0%	69.3%	53.8%	100.0%	69.2%	54.2%
**214**	100.0%	69.3%	53.8%	100.0%	69.2%	54.2%
**215**	100.0%	69.3%	53.8%	100.0%	69.2%	54.2%
**216**	100.0%	69.3%	53.8%	100.0%	69.2%	54.2%
**217**	100.0%	69.3%	53.8%	100.0%	69.2%	54.2%
**218**	100.0%	69.3%	53.8%	100.0%	69.2%	54.2%
**219**	100.0%	69.3%	53.8%	100.0%	69.2%	54.2%
**220**	100.0%	69.3%	53.8%	100.0%	69.2%	54.2%
**221**	100.0%	69.3%	53.8%	100.0%	69.2%	54.2%
**222**	100.0%	69.3%	53.8%	100.0%	69.2%	54.2%
**223**	100.0%	69.3%	53.8%	100.0%	69.2%	54.2%
**224**	100.0%	69.3%	53.8%	100.0%	69.2%	54.2%
**225**	100.0%	69.3%	53.8%	100.0%	69.2%	54.2%
**226**	100.0%	69.3%	53.8%	100.0%	69.2%	54.2%
**227**	100.0%	69.3%	53.8%	100.0%	69.2%	54.2%
**228**	100.0%	69.3%	53.8%	100.0%	69.2%	54.2%
**229**	100.0%	69.3%	53.8%	100.0%	69.2%	54.2%
**230**	100.0%	69.3%	53.8%	100.0%	69.2%	54.2%
**231**	100.0%	69.3%	53.8%	100.0%	69.2%	54.2%
**232**	100.0%	69.3%	53.8%	100.0%	69.2%	54.2%
**233**	100.0%	69.3%	53.8%	100.0%	69.2%	54.2%
**234**	100.0%	69.3%	53.8%	100.0%	69.2%	54.2%
**235**	100.0%	69.3%	53.8%	100.0%	69.2%	54.2%
**236**	100.0%	69.3%	53.8%	100.0%	69.2%	54.2%
**237**	100.0%	69.3%	53.8%	100.0%	69.2%	54.2%
**238**	100.0%	69.3%	53.8%	100.0%	69.2%	54.2%
**239**	100.0%	69.3%	53.8%	100.0%	69.2%	54.2%
**240**	100.0%	69.3%	53.8%	100.0%	69.2%	54.2%

**241**	100.0%	69.3%	53.8%	100.0%	69.2%	54.2%
**242**	100.0%	69.3%	53.8%	100.0%	69.2%	54.2%
**243**	100.0%	69.3%	53.8%	100.0%	69.2%	54.2%
**244**	100.0%	69.3%	53.8%	100.0%	69.2%	54.2%
**245**	100.0%	69.3%	53.8%	100.0%	69.2%	54.2%
**246**	100.0%	69.3%	53.8%	100.0%	69.2%	54.2%
**247**	100.0%	69.3%	53.8%	100.0%	69.2%	54.2%
**248**	100.0%	69.3%	53.8%	100.0%	69.2%	54.2%
**249**	100.0%	69.3%	53.8%	100.0%	69.2%	54.2%
**250**	100.0%	69.3%	53.8%	100.0%	69.2%	54.2%
**251**	100.0%	69.3%	53.8%	100.0%	69.2%	54.2%
**252**	100.0%	69.3%	53.8%	100.0%	69.2%	54.2%
**253**	100.0%	69.3%	53.8%	100.0%	69.2%	54.2%
**254**	100.0%	69.3%	53.8%	100.0%	69.2%	54.2%
**255**	100.0%	69.3%	53.8%	100.0%	69.2%	54.2%
**256**	100.0%	69.3%	53.8%	100.0%	69.2%	54.2%
**257**	100.0%	69.3%	53.8%	100.0%	69.2%	54.2%
**258**	100.0%	69.3%	53.8%	100.0%	69.2%	54.2%
**259**	100.0%	69.3%	53.8%	100.0%	69.2%	54.2%
**260**	100.0%	69.3%	53.8%	100.0%	69.2%	54.2%
**261**	100.0%	69.3%	53.8%	100.0%	69.2%	54.2%
**262**	100.0%	69.3%	53.8%	100.0%	69.2%	54.2%
**263**	100.0%	69.3%	53.8%	100.0%	69.2%	54.2%
**264**	100.0%	69.3%	53.8%	100.0%	69.2%	54.2%
**265**	100.0%	69.3%	53.8%	100.0%	69.2%	54.2%
**266**	100.0%	69.3%	53.8%	100.0%	69.2%	54.2%
**267**	100.0%	69.3%	53.8%	100.0%	69.2%	54.2%
**268**	100.0%	69.3%	53.8%	100.0%	69.2%	54.2%
**269**	100.0%	69.3%	53.8%	100.0%	69.2%	54.2%
**270**	100.0%	69.3%	53.8%	100.0%	69.2%	54.2%
**271**	100.0%	69.3%	53.8%	100.0%	69.2%	54.2%
**272**	100.0%	69.3%	53.8%	100.0%	69.2%	54.2%
**273**	100.0%	69.3%	53.8%	100.0%	69.2%	54.2%
**274**	100.0%	69.3%	53.8%	100.0%	69.2%	54.2%
**275**	100.0%	69.3%	53.8%	100.0%	69.2%	54.2%
**276**	100.0%	69.3%	53.8%	100.0%	69.2%	54.2%
**277**	100.0%	69.3%	53.8%	100.0%	69.2%	54.2%
**278**	100.0%	69.3%	53.8%	100.0%	69.2%	54.2%
**279**	100.0%	69.3%	53.8%	100.0%	69.2%	54.2%
**280**	100.0%	69.3%	53.8%	100.0%	69.2%	54.2%
**281**	100.0%	69.3%	53.8%	100.0%	69.2%	54.2%
**282**	100.0%	69.3%	53.8%	100.0%	69.2%	54.2%
**283**	100.0%	69.3%	53.8%	100.0%	69.2%	54.2%
**284**	100.0%	69.3%	53.8%	100.0%	69.2%	54.2%
**285**	100.0%	69.3%	53.8%	100.0%	69.2%	54.2%
**286**	100.0%	69.3%	53.8%	100.0%	69.2%	54.2%
**287**	100.0%	69.3%	53.8%	100.0%	69.2%	54.2%

**288**	100.0%	69.3%	53.8%	100.0%	69.2%	54.2%
**289**	100.0%	69.3%	53.8%	100.0%	69.2%	54.2%
**290**	100.0%	69.3%	53.8%	100.0%	69.2%	54.2%
**291**	100.0%	69.3%	53.8%	100.0%	69.2%	54.2%
**292**	100.0%	69.3%	53.8%	100.0%	69.2%	54.2%
**293**	100.0%	69.3%	53.8%	100.0%	69.2%	54.2%
**294**	100.0%	69.3%	53.8%	100.0%	69.2%	54.2%
**295**	100.0%	69.3%	53.8%	100.0%	69.2%	54.2%
**296**	100.0%	69.3%	53.8%	100.0%	69.2%	54.2%
**297**	100.0%	69.3%	53.8%	100.0%	69.2%	54.2%
**298**	100.0%	69.3%	53.8%	100.0%	69.2%	54.2%
**299**	100.0%	69.3%	53.8%	100.0%	69.2%	54.2%
**300**	100.0%	69.3%	53.8%	100.0%	69.2%	54.2%
**301**	100.0%	69.3%	53.8%	100.0%	69.2%	54.2%
**302**	100.0%	69.3%	53.8%	100.0%	69.2%	54.2%
**303**	100.0%	69.3%	53.8%	100.0%	69.2%	54.2%
**304**	100.0%	69.3%	53.8%	100.0%	69.2%	54.2%
**305**	100.0%	69.3%	53.8%	100.0%	69.2%	54.2%
**306**	100.0%	69.3%	53.8%	100.0%	69.2%	54.2%
**307**	100.0%	69.3%	53.8%	100.0%	69.2%	54.2%
**308**	100.0%	69.3%	53.8%	100.0%	69.2%	54.2%
**309**	100.0%	69.3%	53.8%	100.0%	69.2%	54.2%
**310**	100.0%	69.3%	53.8%	100.0%	69.2%	54.2%
**311**	100.0%	69.3%	53.8%	100.0%	69.2%	54.2%
**312**	100.0%	69.3%	53.8%	100.0%	69.2%	54.2%
**313**	100.0%	69.3%	53.8%	100.0%	69.2%	54.2%
**314**	100.0%	69.3%	53.8%	100.0%	69.2%	54.2%
**315**	100.0%	69.3%	53.8%	100.0%	69.2%	54.2%
**316**	100.0%	69.3%	53.8%	100.0%	69.2%	54.2%
**317**	100.0%	69.3%	53.8%	100.0%	69.2%	54.2%
**318**	100.0%	69.3%	53.8%	100.0%	69.2%	54.2%
**319**	100.0%	69.3%	53.8%	100.0%	69.2%	54.2%
**320**	100.0%	69.3%	53.8%	100.0%	69.2%	54.2%
**321**	100.0%	69.3%	53.8%	100.0%	69.2%	54.2%
**322**	100.0%	69.3%	53.8%	100.0%	69.2%	54.2%
**323**	100.0%	69.3%	53.8%	100.0%	69.2%	54.2%
**324**	100.0%	69.3%	53.8%	100.0%	69.2%	54.2%
**325**	100.0%	69.3%	53.8%	100.0%	69.2%	54.2%
**326**	100.0%	69.3%	53.8%	100.0%	69.2%	54.2%
**327**	100.0%	69.3%	53.8%	100.0%	69.2%	54.2%
**328**	100.0%	69.3%	53.8%	100.0%	69.2%	54.2%
**329**	100.0%	69.3%	53.8%	100.0%	69.2%	54.2%
**330**	100.0%	69.3%	53.8%	100.0%	69.2%	54.2%
**331**	100.0%	69.3%	53.8%	100.0%	69.2%	54.2%
**332**	100.0%	69.3%	53.8%	100.0%	69.2%	54.2%
**333**	100.0%	69.3%	53.8%	100.0%	69.2%	54.2%
**334**	100.0%	69.3%	53.8%	100.0%	69.2%	54.2%

**335**	100.0%	69.3%	53.8%	100.0%	69.2%	54.2%
**336**	100.0%	69.3%	53.8%	100.0%	69.2%	54.2%
**337**	100.0%	69.3%	53.8%	100.0%	69.2%	54.2%
**338**	100.0%	69.3%	53.8%	100.0%	69.2%	54.2%
**339**	100.0%	69.3%	53.8%	100.0%	69.2%	54.2%
**340**	100.0%	69.3%	53.8%	100.0%	69.2%	54.2%
**341**	100.0%	69.3%	53.8%	100.0%	69.2%	54.2%
**342**	100.0%	69.3%	53.8%	100.0%	69.2%	54.2%
**343**	100.0%	69.3%	53.8%	100.0%	69.2%	54.2%
**344**	100.0%	69.3%	53.8%	100.0%	69.2%	54.2%
**345**	100.0%	69.3%	53.8%	100.0%	69.2%	54.2%
**346**	100.0%	69.3%	53.8%	100.0%	69.2%	54.2%
**347**	100.0%	69.3%	53.8%	100.0%	69.2%	54.2%
**348**	100.0%	69.3%	53.8%	100.0%	69.2%	54.2%
**349**	100.0%	69.3%	53.8%	100.0%	69.2%	54.2%
**350**	100.0%	69.3%	53.8%	100.0%	69.2%	54.2%
**351**	100.0%	69.3%	53.8%	100.0%	69.2%	54.2%
**352**	100.0%	69.3%	53.8%	100.0%	69.2%	54.2%
**353**	100.0%	69.3%	53.8%	100.0%	69.2%	54.2%
**354**	100.0%	69.3%	53.8%	100.0%	69.2%	54.2%
**355**	100.0%	69.3%	53.8%	100.0%	69.2%	54.2%
**356**	100.0%	69.3%	53.8%	100.0%	69.2%	54.2%
**357**	100.0%	69.3%	53.8%	100.0%	69.2%	54.2%
**358**	100.0%	69.3%	53.8%	100.0%	69.2%	54.2%
**359**	100.0%	69.3%	53.8%	100.0%	69.2%	54.2%
**360**	100.0%	69.3%	53.8%	100.0%	69.2%	54.2%
**361**	100.0%	69.3%	53.8%	100.0%	69.2%	54.2%
**362**	100.0%	69.3%	53.8%	100.0%	69.2%	54.2%
**363**	100.0%	69.3%	53.8%	100.0%	69.2%	54.2%
**364**	100.0%	69.3%	53.8%	100.0%	69.2%	54.2%
