# Supplementary material for: Effects of Tobacco Smoking on the Degeneration of the Intervertebral Disc: A Finite Element Study
Source: PLoS One. 2015 Aug 24;10(8):e0136137. doi: 10.1371/journal.pone.0136137 (PMC4547737; doi:10.1371/journal.pone.0136137)
Supplement: S5 File — GAG levels and cell density in all disc regions are reported for both ‘light smoking’ and ‘heavy smoking’ scenarios. Data are normalized with respect to ‘non-smoking’ scenario. (PDF) [file pone.0136137.s005.pdf]

	Cell		Gag	
day	heavy	light	heavy	light
0	91.9%	111.0%	72.7%	81.9%
1	99.9%	120.5%	101.1%	121.8%
2	92.3%	115.6%	93.5%	116.8%
3	86.2%	110.5%	87.2%	111.6%
4	81.1%	105.3%	82.0%	106.3%
5	80.1%	99.8%	81.1%	100.8%
6	79.7%	95.7%	80.6%	96.6%
7	79.5%	95.3%	80.4%	96.1%
8	79.5%	95.2%	80.4%	96.1%
9	79.3%	94.9%	80.2%	95.8%
10	79.2%	94.8%	80.2%	95.6%
11	79.2%	94.6%	80.1%	95.5%
12	79.1%	94.6%	80.1%	95.4%
13	79.0%	94.6%	80.0%	95.4%
14	79.0%	94.5%	79.9%	95.4%
15	79.0%	94.4%	79.9%	95.3%
16	79.0%	94.4%	79.9%	95.2%
17	79.0%	94.4%	79.9%	95.2%
18	79.0%	94.4%	79.9%	95.2%
19	79.0%	94.4%	79.9%	95.2%
20	79.0%	94.4%	79.9%	95.2%
21	79.0%	94.3%	79.9%	95.2%
22	78.9%	94.3%	79.8%	95.2%
23	78.9%	94.3%	79.8%	95.2%
24	78.9%	94.3%	79.8%	95.2%
25	78.9%	94.3%	79.8%	95.2%
26	78.9%	94.3%	79.8%	95.2%
27	78.9%	94.3%	79.8%	95.2%
28	78.9%	94.3%	79.8%	95.2%
29	78.9%	94.3%	79.8%	95.2%
30	78.9%	94.3%	79.8%	95.1%
31	78.9%	94.3%	79.8%	95.1%
32	78.9%	94.3%	79.8%	95.1%
33	78.9%	94.3%	79.8%	95.1%
34	78.9%	94.3%	79.8%	95.1%
35	78.9%	94.3%	79.8%	95.1%
36	78.9%	94.3%	79.8%	95.1%
37	78.9%	94.3%	79.8%	95.1%
38	78.9%	94.3%	79.8%	95.1%
39	78.9%	94.3%	79.8%	95.1%
40	78.9%	94.3%	79.8%	95.1%
41	78.9%	94.3%	79.8%	95.1%
42	78.9%	94.3%	79.8%	95.1%
43	78.9%	94.3%	79.8%	95.1%
44	78.9%	94.3%	79.8%	95.1%

**45**	78.9%	94.3%		79.8%	95.1%
**46**	78.9%	94.3%		79.8%	95.1%
**47**	78.9%	94.3%		79.8%	95.1%
**48**	78.9%	94.3%		79.8%	95.1%
**49**	78.9%	94.3%		79.7%	95.1%
**50**	78.9%	94.3%		79.7%	95.1%
**51**	78.9%	94.3%		79.7%	95.1%
**52**	78.9%	94.3%		79.7%	95.1%
**53**	78.9%	94.3%		79.7%	95.1%
**54**	78.9%	94.3%		79.7%	95.1%
**55**	78.9%	94.3%		79.7%	95.1%
**56**	78.9%	94.3%		79.7%	95.1%
**57**	78.9%	94.3%		79.7%	95.1%
**58**	78.9%	94.3%		79.7%	95.1%
**59**	79.0%	94.3%		79.7%	95.1%
**60**	79.0%	94.3%		79.7%	95.1%
**61**	79.0%	94.3%		79.7%	95.1%
**62**	79.0%	94.3%		79.7%	95.1%
**63**	79.0%	94.3%		79.7%	95.1%
**64**	79.0%	94.3%		79.7%	95.1%
**65**	79.0%	94.3%		79.7%	95.1%
**66**	79.0%	94.3%		79.7%	95.1%
**67**	79.0%	94.3%		79.7%	95.1%
**68**	79.0%	94.3%		79.7%	95.1%
**69**	79.0%	94.3%		79.7%	95.1%
**70**	79.0%	94.3%		79.7%	95.1%
**71**	79.0%	94.3%		79.7%	95.1%
**72**	79.0%	94.3%		79.7%	95.1%
**73**	79.0%	94.3%		79.7%	95.1%
**74**	79.0%	94.3%		79.7%	95.0%
**75**	79.0%	94.3%		79.7%	95.0%
**76**	79.0%	94.3%		79.7%	95.0%
**77**	79.0%	94.3%		79.7%	95.0%
**78**	79.0%	94.3%		79.7%	95.0%
**79**	79.0%	94.3%		79.7%	95.0%
**80**	79.0%	94.3%		79.7%	95.0%
**81**	79.0%	94.3%		79.7%	95.0%
**82**	79.0%	94.3%		79.7%	95.0%
**83**	79.0%	94.3%		79.7%	95.0%
**84**	79.0%	94.3%		79.7%	95.0%
**85**	79.0%	94.3%		79.7%	95.0%
**86**	79.0%	94.3%		79.7%	95.0%
**87**	79.0%	94.3%		79.7%	95.0%
**88**	79.0%	94.3%		79.7%	95.0%
**89**	79.0%	94.3%		79.7%	95.0%
**90**	79.0%	94.3%		79.7%	95.0%
**91**	79.0%	94.3%		79.7%	95.0%

**92**	79.0%	94.3%	79.7%	95.0%
**93**	79.0%	94.3%	79.7%	95.0%
**94**	79.0%	94.3%	79.7%	95.0%
**95**	79.0%	94.3%	79.7%	95.0%
**96**	79.0%	94.3%	79.7%	95.0%
**97**	79.0%	94.3%	79.7%	95.0%
**98**	79.0%	94.3%	79.7%	95.0%
**99**	79.0%	94.3%	79.7%	95.0%
**100**	79.0%	94.3%	79.7%	95.0%
**101**	79.0%	94.3%	79.7%	95.0%
**102**	79.0%	94.4%	79.7%	95.0%
**103**	79.0%	94.4%	79.7%	95.0%
**104**	79.0%	94.3%	79.7%	95.0%
**105**	79.0%	94.4%	79.7%	95.0%
**106**	79.0%	94.4%	79.7%	95.0%
**107**	79.0%	94.4%	79.7%	95.0%
**108**	79.0%	94.4%	79.7%	95.0%
**109**	79.0%	94.4%	79.7%	95.0%
**110**	79.0%	94.4%	79.7%	95.0%
**111**	79.0%	94.4%	79.7%	95.0%
**112**	79.0%	94.4%	79.7%	95.0%
**113**	79.0%	94.4%	79.7%	95.0%
**114**	79.0%	94.4%	79.7%	95.0%
**115**	79.0%	94.4%	79.7%	95.0%
**116**	79.0%	94.4%	79.7%	95.0%
**117**	79.0%	94.4%	79.7%	95.0%
**118**	79.0%	94.4%	79.7%	95.0%
**119**	79.0%	94.4%	79.7%	95.0%
**120**	79.0%	94.4%	79.7%	95.0%
**121**	79.0%	94.4%	79.7%	95.0%
**122**	79.0%	94.4%	79.7%	95.0%
**123**	79.0%	94.4%	79.7%	95.0%
**124**	79.0%	94.4%	79.7%	95.0%
**125**	79.0%	94.4%	79.7%	95.0%
**126**	79.0%	94.4%	79.7%	95.0%
**127**	79.0%	94.4%	79.7%	95.0%
**128**	79.0%	94.4%	79.7%	95.0%
**129**	79.0%	94.4%	79.7%	95.0%
**130**	79.0%	94.4%	79.7%	95.0%
**131**	79.0%	94.4%	79.7%	95.0%
**132**	79.0%	94.4%	79.7%	95.0%
**133**	79.0%	94.4%	79.7%	95.0%
**134**	79.0%	94.4%	79.7%	95.0%
**135**	79.0%	94.4%	79.7%	95.0%
**136**	79.0%	94.4%	79.7%	95.0%
**137**	79.0%	94.4%	79.7%	95.0%
**138**	79.0%	94.4%	79.7%	95.0%

**139**	79.0%	94.4%	79.7%	95.0%
**140**	79.0%	94.4%	79.7%	95.0%
**141**	79.0%	94.4%	79.7%	95.0%
**142**	79.0%	94.4%	79.7%	95.0%
**143**	79.0%	94.4%	79.7%	95.0%
**144**	79.0%	94.4%	79.7%	95.0%
**145**	79.0%	94.4%	79.7%	95.0%
**146**	79.0%	94.4%	79.7%	95.0%
**147**	79.0%	94.4%	79.7%	95.0%
**148**	79.0%	94.4%	79.7%	95.0%
**149**	79.0%	94.4%	79.7%	95.0%
**150**	79.0%	94.4%	79.7%	95.0%
**151**	79.0%	94.4%	79.7%	95.0%
**152**	79.0%	94.4%	79.7%	95.0%
**153**	79.0%	94.4%	79.7%	95.0%
**154**	79.0%	94.4%	79.7%	95.0%
**155**	79.0%	94.4%	79.7%	95.0%
**156**	79.0%	94.4%	79.7%	95.0%
**157**	79.0%	94.4%	79.7%	95.0%
**158**	79.0%	94.4%	79.7%	95.0%
**159**	79.0%	94.4%	79.7%	95.0%
**160**	79.0%	94.4%	79.7%	95.0%
**161**	79.0%	94.4%	79.7%	95.0%
**162**	79.0%	94.4%	79.7%	95.0%
**163**	79.0%	94.4%	79.7%	95.0%
**164**	79.0%	94.4%	79.7%	95.0%
**165**	79.0%	94.4%	79.7%	95.0%
**166**	79.0%	94.4%	79.7%	95.0%
**167**	79.0%	94.4%	79.7%	95.0%
**168**	79.0%	94.4%	79.7%	95.0%
**169**	79.0%	94.4%	79.7%	95.0%
**170**	79.0%	94.4%	79.7%	95.0%
**171**	79.0%	94.4%	79.7%	95.0%
**172**	79.0%	94.4%	79.7%	95.0%
**173**	79.0%	94.4%	79.7%	95.0%
**174**	79.0%	94.4%	79.7%	95.0%
**175**	79.0%	94.4%	79.7%	95.0%
**176**	79.0%	94.4%	79.7%	95.0%
**177**	79.0%	94.4%	79.7%	95.0%
**178**	79.0%	94.4%	79.7%	95.0%
**179**	79.0%	94.4%	79.7%	95.0%
**180**	79.0%	94.4%	79.7%	95.0%
**181**	79.0%	94.4%	79.7%	95.0%
**182**	79.0%	94.4%	79.7%	95.0%
**183**	79.0%	94.4%	79.7%	95.0%
**184**	79.0%	94.4%	79.7%	95.0%
**185**	79.0%	94.4%	79.7%	95.0%

**186**	79.0%	94.4%	79.7%	95.0%
**187**	79.0%	94.4%	79.7%	95.0%
**188**	79.0%	94.4%	79.7%	95.0%
**189**	79.0%	94.4%	79.7%	95.0%
**190**	79.0%	94.4%	79.7%	95.0%
**191**	79.0%	94.4%	79.7%	95.0%
**192**	79.0%	94.4%	79.7%	95.0%
**193**	79.0%	94.4%	79.7%	95.0%
**194**	79.0%	94.4%	79.7%	95.0%
**195**	79.0%	94.4%	79.7%	95.0%
**196**	79.0%	94.4%	79.7%	95.0%
**197**	79.0%	94.4%	79.7%	95.0%
**198**	79.0%	94.4%	79.7%	95.0%
**199**	79.0%	94.4%	79.7%	95.0%
**200**	79.0%	94.4%	79.7%	95.0%
**201**	79.0%	94.4%	79.7%	95.0%
**202**	79.0%	94.4%	79.7%	95.0%
**203**	79.0%	94.4%	79.7%	95.0%
**204**	79.0%	94.4%	79.7%	95.0%
**205**	79.0%	94.4%	79.7%	95.0%
**206**	79.0%	94.4%	79.7%	95.0%
**207**	79.0%	94.4%	79.7%	95.0%
**208**	79.0%	94.4%	79.7%	95.0%
**209**	79.0%	94.4%	79.7%	95.0%
**210**	79.0%	94.4%	79.7%	95.0%
**211**	79.0%	94.4%	79.7%	95.0%
**212**	79.0%	94.4%	79.7%	95.0%
**213**	79.0%	94.4%	79.7%	95.0%
**214**	79.0%	94.4%	79.7%	95.0%
**215**	79.0%	94.4%	79.7%	95.0%
**216**	79.0%	94.4%	79.7%	95.0%
**217**	79.0%	94.4%	79.7%	95.0%
**218**	79.0%	94.4%	79.7%	95.0%
**219**	79.0%	94.4%	79.7%	95.0%
**220**	79.0%	94.4%	79.7%	95.0%
**221**	79.0%	94.4%	79.7%	95.0%
**222**	79.0%	94.4%	79.7%	95.0%
**223**	79.0%	94.4%	79.7%	95.0%
**224**	79.0%	94.4%	79.7%	95.0%
**225**	79.0%	94.4%	79.7%	95.0%
**226**	79.0%	94.4%	79.7%	95.0%
**227**	79.0%	94.4%	79.7%	95.0%
**228**	79.0%	94.4%	79.7%	95.0%
**229**	79.0%	94.4%	79.7%	95.0%
**230**	79.0%	94.4%	79.7%	95.0%
**231**	79.0%	94.4%	79.7%	95.0%
**232**	79.0%	94.4%	79.7%	95.0%

**233**	79.0%	94.4%		79.7%	95.0%
**234**	79.0%	94.4%		79.7%	95.0%
**235**	79.0%	94.4%		79.7%	95.0%
**236**	79.0%	94.4%		79.7%	95.0%
**237**	79.0%	94.4%		79.7%	95.0%
**238**	79.0%	94.4%		79.7%	95.0%
**239**	79.0%	94.4%		79.7%	95.0%
**240**	79.0%	94.4%		79.7%	95.0%
**241**	79.0%	94.4%		79.7%	95.0%
**242**	79.0%	94.4%		79.7%	95.0%
**243**	79.0%	94.4%		79.7%	95.0%
**244**	79.0%	94.4%		79.7%	95.0%
**245**	79.0%	94.4%		79.7%	95.0%
**246**	79.0%	94.4%		79.7%	95.0%
**247**	79.0%	94.4%		79.7%	95.0%
**248**	79.0%	94.4%		79.7%	95.0%
**249**	79.0%	94.4%		79.7%	95.0%
**250**	79.0%	94.4%		79.7%	95.0%
**251**	79.0%	94.4%		79.7%	95.0%
**252**	79.0%	94.4%		79.7%	95.0%
**253**	79.0%	94.4%		79.7%	95.0%
**254**	79.0%	94.4%		79.7%	95.0%
**255**	79.0%	94.4%		79.7%	95.0%
**256**	79.0%	94.4%		79.7%	95.0%
**257**	79.0%	94.4%		79.7%	95.0%
**258**	79.0%	94.4%		79.7%	95.0%
**259**	79.0%	94.4%		79.7%	95.0%
**260**	79.0%	94.4%		79.7%	95.0%
**261**	79.0%	94.4%		79.7%	95.0%
**262**	79.0%	94.4%		79.7%	95.0%
**263**	79.0%	94.4%		79.7%	95.0%
**264**	79.0%	94.4%		79.7%	95.0%
**265**	79.0%	94.4%		79.7%	95.0%
**266**	79.0%	94.4%		79.7%	95.0%
**267**	79.0%	94.4%		79.7%	95.0%
**268**	79.0%	94.4%		79.7%	95.0%
**269**	79.0%	94.4%		79.7%	95.0%
**270**	79.0%	94.4%		79.7%	95.0%
**271**	79.0%	94.4%		79.7%	95.0%
**272**	79.0%	94.4%		79.7%	95.0%
**273**	79.0%	94.4%		79.7%	95.0%
**274**	79.0%	94.4%		79.7%	95.0%
**275**	79.0%	94.4%		79.7%	95.0%
**276**	79.0%	94.4%		79.7%	95.0%
**277**	79.0%	94.4%		79.7%	95.0%
**278**	79.0%	94.4%		79.7%	95.0%
**279**	79.0%	94.4%		79.7%	95.0%

**280**	79.0%	94.4%	79.7%	95.0%
**281**	79.0%	94.4%	79.7%	95.0%
**282**	79.0%	94.4%	79.7%	95.0%
**283**	79.0%	94.4%	79.7%	95.0%
**284**	79.0%	94.4%	79.7%	95.0%
**285**	79.0%	94.4%	79.7%	95.0%
**286**	79.0%	94.4%	79.7%	95.0%
**287**	79.0%	94.4%	79.7%	95.0%
**288**	79.0%	94.4%	79.7%	95.0%
**289**	79.0%	94.4%	79.7%	95.0%
**290**	79.0%	94.4%	79.7%	95.0%
**291**	79.0%	94.4%	79.7%	95.0%
**292**	79.0%	94.4%	79.7%	95.0%
**293**	79.0%	94.4%	79.7%	95.0%
**294**	79.0%	94.4%	79.7%	95.0%
**295**	79.0%	94.4%	79.7%	95.0%
**296**	79.0%	94.4%	79.7%	95.0%
**297**	79.0%	94.4%	79.7%	95.0%
**298**	79.0%	94.4%	79.7%	95.0%
**299**	79.0%	94.4%	79.7%	95.0%
**300**	79.0%	94.4%	79.7%	95.0%
**301**	79.0%	94.4%	79.7%	95.0%
**302**	79.0%	94.4%	79.7%	95.0%
**303**	79.0%	94.4%	79.7%	95.0%
**304**	79.0%	94.4%	79.7%	95.0%
**305**	79.0%	94.4%	79.7%	95.0%
**306**	79.0%	94.4%	79.7%	95.0%
**307**	79.0%	94.4%	79.7%	95.0%
**308**	79.0%	94.4%	79.7%	95.0%
**309**	79.0%	94.4%	79.7%	95.0%
**310**	79.0%	94.4%	79.7%	95.0%
**311**	79.0%	94.4%	79.7%	95.0%
**312**	79.0%	94.4%	79.7%	95.0%
**313**	79.0%	94.4%	79.7%	95.0%
**314**	79.0%	94.4%	79.7%	95.0%
**315**	79.0%	94.4%	79.7%	95.0%
**316**	79.0%	94.4%	79.7%	95.0%
**317**	79.0%	94.4%	79.7%	95.0%
**318**	79.0%	94.4%	79.7%	95.0%
**319**	79.0%	94.4%	79.7%	95.0%
**320**	79.0%	94.4%	79.7%	95.0%
**321**	79.0%	94.4%	79.7%	95.0%
**322**	79.0%	94.4%	79.7%	95.0%
**323**	79.0%	94.4%	79.7%	95.0%
**324**	79.0%	94.4%	79.7%	95.0%
**325**	79.0%	94.4%	79.7%	95.0%
**326**	79.0%	94.4%	79.7%	95.0%

**327**	79.0%	94.4%		79.7%	95.0%
**328**	79.0%	94.4%		79.7%	95.0%
**329**	79.0%	94.4%		79.7%	95.0%
**330**	79.0%	94.4%		79.7%	95.0%
**331**	79.0%	94.4%		79.7%	95.0%
**332**	79.0%	94.4%		79.7%	95.0%
**333**	79.0%	94.4%		79.7%	95.0%
**334**	79.0%	94.4%		79.7%	95.0%
**335**	79.0%	94.4%		79.7%	95.0%
**336**	79.0%	94.4%		79.7%	95.0%
**337**	79.0%	94.4%		79.7%	95.0%
**338**	79.0%	94.4%		79.7%	95.0%
**339**	79.0%	94.4%		79.7%	95.0%
**340**	79.0%	94.4%		79.7%	95.0%
**341**	79.0%	94.4%		79.7%	95.0%
**342**	79.0%	94.4%		79.7%	95.0%
**343**	79.0%	94.4%		79.7%	95.0%
**344**	79.0%	94.4%		79.7%	95.0%
**345**	79.0%	94.4%		79.7%	95.0%
**346**	79.0%	94.4%		79.7%	95.0%
**347**	79.0%	94.4%		79.7%	95.0%
**348**	79.0%	94.4%		79.7%	95.0%
**349**	79.0%	94.4%		79.7%	95.0%
**350**	79.0%	94.4%		79.7%	95.0%
**351**	79.0%	94.4%		79.7%	95.0%
**352**	79.0%	94.4%		79.7%	95.0%
**353**	79.0%	94.4%		79.7%	95.0%
**354**	79.0%	94.4%		79.7%	95.0%
**355**	79.0%	94.4%		79.7%	95.0%
**356**	79.0%	94.4%		79.7%	95.0%
**357**	79.0%	94.4%		79.7%	95.0%
**358**	79.0%	94.4%		79.7%	95.0%
**359**	79.0%	94.4%		79.7%	95.0%
**360**	79.0%	94.4%		79.7%	95.0%
**361**	79.0%	94.4%		79.7%	95.0%
**362**	79.0%	94.4%		79.7%	95.0%
**363**	79.0%	94.4%		79.7%	95.0%
**364**	79.0%	94.4%		79.7%	95.0%
